# Failure to Burrow and Tunnel Reveals Roles for *jim lovell* in the Growth and Endoreplication of the Drosophila Larval Tracheae

**DOI:** 10.1371/journal.pone.0160233

**Published:** 2016-08-05

**Authors:** Fanli Zhou, Karen M. Qiang, Kathleen M. Beckingham

**Affiliations:** Department of Biosciences, Rice University, Houston, Texas, 77005, United States of America; University of Mississippi, UNITED STATES

## Abstract

The Drosophila protein Jim Lovell (Lov) is a putative transcription factor of the BTB/POZ (**B**ric- a-Brac/**T**ramtrack/**B**road/ **Po**x virus and **Z**inc finger) domain class that is expressed in many elements of the developing larval nervous system. It has roles in innate behaviors such as larval locomotion and adult courtship. In performing tissue-specific knockdown with the Gal4-UAS system we identified a new behavioral phenotype for *lov*: larvae failed to burrow into their food during their growth phase and then failed to tunnel into an agarose substratum during their wandering phase. We determined that these phenotypes originate in a previously unrecognized role for *lov* in the tracheae. By using tracheal-specific Gal4 lines, Lov immunolocalization and a *lov* enhancer trap line, we established that *lov* is normally expressed in the tracheae from late in embryogenesis through larval life. Using an assay that monitors food burrowing, substrate tunneling and death we showed that *lov* tracheal knockdown results in tracheal fluid-filling, producing hypoxia that activates the aberrant behaviors and inhibits development. We investigated the role of *lov* in the tracheae that initiates this sequence of events. We discovered that when *lov* levels are reduced, the tracheal cells are smaller, more numerous and show lower levels of endopolyploidization. Together our findings indicate that Lov is necessary for tracheal endoreplicative growth and that its loss in this tissue causes loss of tracheal integrity resulting in chronic hypoxia and abnormal burrowing and tunneling behavior.

## Introduction

During their rapid growth period, Drosophila larvae burrow into their food, keeping only their posterior spiracles exposed at the food surface. As they transition from feeding to pupation, the larvae undertake a wandering phase in which they move away from food and seek out a site to settle for the prepupal and pupal molts. This wandering phase is a vulnerable period in the life cycle when exposure to environmental risks such as desiccation or predators can occur [1, 2]. Previous studies have established that wandering larvae will dig through a relatively soft substratum, creating tunnels, rather than moving across the surface [3]. This behavior may thus represent an innate defensive tactic. In general, there has been little analysis of this tunneling behavior, although a recent study demonstrated that it is subject to evolutionary modification [4]. Unexpectedly, in pursuing the roles of the Drosophila gene *jim lovell* (*lov*) we have discovered a link between this gene and larval burrowing and tunneling behaviors.

Our prior studies have already identified roles for *lov* in other larval and adult behaviors [5, 6] and suggested routes by which *lov* might affect these responses. *lov* encodes a putative transcription factor of the BTB/POZ domain family [7] and immunolocalization studies in embryogenesis established that Lov is expressed in the nuclei of many subsets of neurons in the PNS and CNS late in their development [6]. It seems probable therefore that *lov* acts to specify the terminal differentiation of particular classes of neurons and that its roles in behavior reflect these functions.

In order to dissect out the individual roles of *lov* in different subsets of neurons we undertook neuronal cell-type specific knockdown of *lov*, using the Gal4 system [8] in combination with *lov* RNAi. A series of neuron-specific Gal4 lines were screened for their effects on development and behavior when driving *lov* RNAi. Given that *lov* is expressed in almost all of the external sense organ (eso) neurons of the embryonic PNS we particularly sought Gal4 drivers that would express *lov* RNAi uniquely in eso lineages. The transcription factor Cut is essential for the maintenance of eso lineages [9] in both the embryo and the adult wing margin [10]. Regions of the *cut* locus that drive expression in the embryonic esos have been identified [11], but no *cut-*Gal4 driver that expresses specifically in these esos has been generated. However, a *cut-*Gal4 driver that directs transcription in the esos of the adult wing margin is available [10, 12] (Blochlinger, communication to Flybase FBrf0125080). This driver, termed here *cut(ue)-*Gal4 to denote the *cut* **u**pstream **e**nhancer it contains, was therefore included in our screen.

In contrast to the other Gal4 lines tested with *lov* RNAi, *cut(ue)-*Gal4 produced partial lethality in the larval phase that proved to be associated with an absence of burrowing and tunneling behaviors. In investigating these behaviors we uncovered a new role for *lov* in the growth of the larval tracheae that appears to be the origin of these behavioral defects. The damage produced by tracheal *lov* knockdown leads to hypoxia, which has been shown previously to cause larvae to exit their food burrows [13]. We show here that substratum tunneling in the wandering phase is also inhibited by hypoxia, thus establishing that both behaviors reflect the tracheal function of *lov*.

## Results

### Co-expression of *cut(ue)-*Gal4 and *lov* in the tracheal epithelial cells

Three *lov* RNAi lines were available from the Drosophila RNAi Consortia (two from the TRiP Center at Harvard Medical School and one from the Vienna Drosophila RNAi Center) for these studies. We used Semi-Q RT-PCR to assess the effectiveness of these constructs at depleting *lov* RNA in the late embryonic nervous system when driven by the *elav*-Gal4 driver (S1 Fig). The Valium 20 *lov* RNAi construct (HMSO01126) from the TRiP facility proved most effective and was used as the *lov* RNAi construct for all the studies described here.

**Fig 1:**
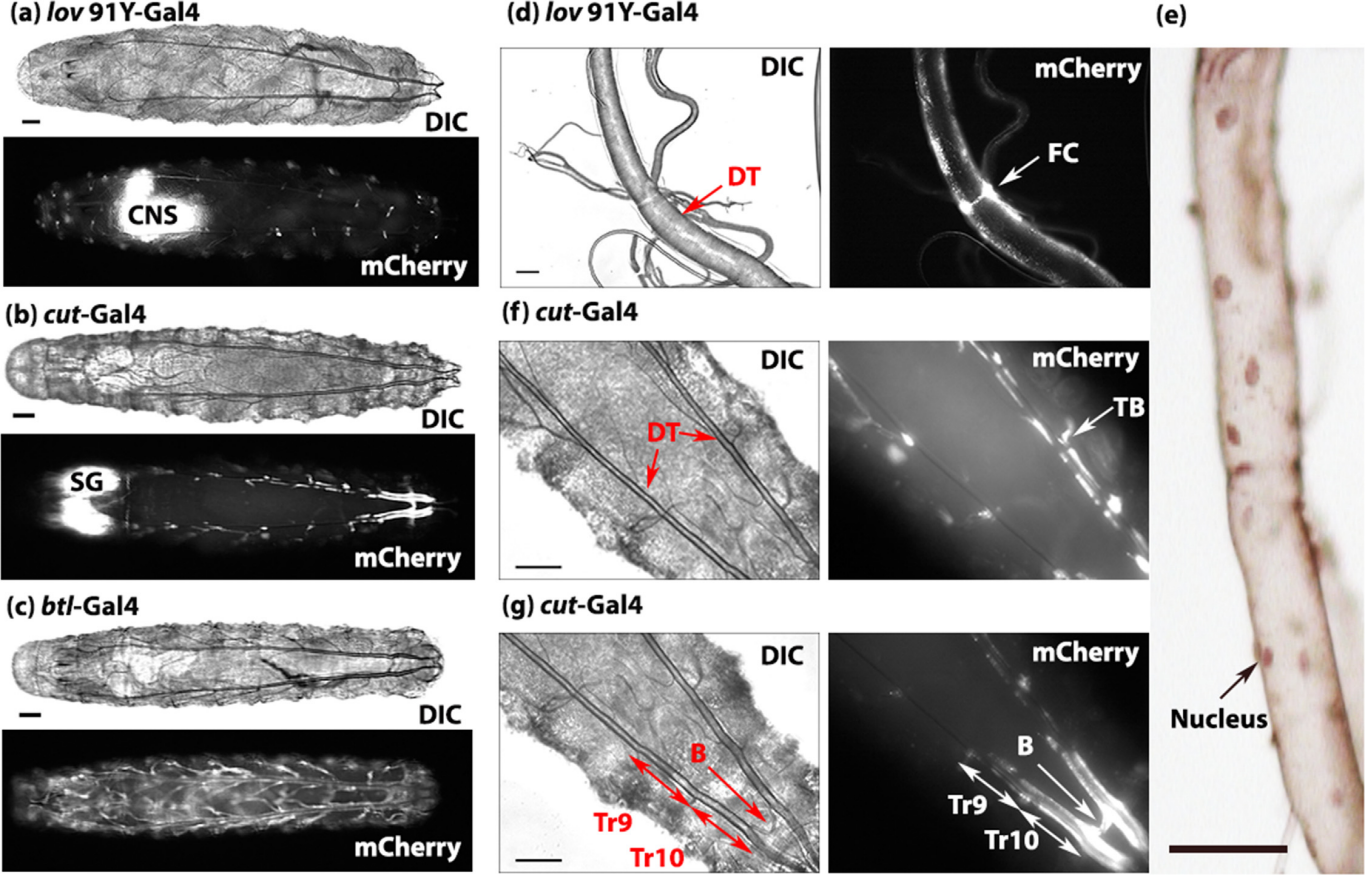
Larval expression patterns of the Gal4 drivers used in these studies. (a-c) DIC and fluorescence images of first instar larvae expressing UAS-mCherry driven by *lov^91Y^*-Gal4 (a), *cut(ue)*-Gal4 (b), and *btl*-Gal4 (c). Identical exposure conditions were used for the fluorescence images to reveal the differences in expression levels for the three Gal4 lines. *lov^91Y^*-Gal4 shows neural expression and faint activity in the tracheal DT epithelial cells. The bright dots of fluorescence in (a) are either fusion cells of the DTs or nuclei of peripheral neurons. (d) shows *lov^91Y^*-Gal4 expression in the epithelial cells and fusion cells (FC) of a third instar larval DT, (e) shows Lov immunostaining in DT nuclei at the same larval stage, (f) shows expression of *cut(ue)*-Gal4 in a cluster of tracheoblast cells (TB) in a first instar larvae, (g) shows high expression of *cut(ue)*-Gal4 in Tr10 of the DTs, the bridge (B), and the posterior section of the DTs adjacent to the spiracles. Scale bars = 50 μm. CNS = central nervous system, DT = dorsal trunks, SG = salivary glands.

We initially screened for effects of *lov* RNAi driven by multiple neuronal drivers, and all but one produced behavioral anomalies (such as defective larval locomotion or adult courtship) without affecting overall viability. The unique lethality seen for *cut(ue)-*Gal4 > *lov* RNAi animals was therefore investigated further, beginning with a characterization of the early *cut(ue)-*Gal4 expression pattern. Using UAS-mCherry as a reporter [14], we determined that, in contrast to our expectation, this driver shows no neural expression in embryonic or larval life, but rather the Gal4 pattern is entirely limited to the epithelial cells of the tracheal system and the attached tracheoblasts (Fig 1B and 1F). The tracheoblasts are clusters of cells destined to generate elements of the adult tracheal system [15]. Further, the Gal4 expression under *cut(ue)-*Gal4 is highly regional within the tracheal dorsal trunks (DTs). It is largely limited to the three posterior sections, with weak expression in metamere Tr9 and very strong expression in i) metamere Tr10, ii) the connection between the DTs posterior to Tr10 (termed here the “bridge”), and iii) the final segment of the DTs adjacent to the spiracles (Fig 1B and 1G). Cut is a homeobox transcription factor that functions in the differentiation of many cell types and derives from a complex locus spanning 200 kb on the X chromosome (7B4-6) [11, 16]. Its role in the larval tracheae and tracheoblasts has been studied previously [17–19] and one *cut-*Gal4 enhancer trap line (PG142) that gives Gal4 expression in the tracheae and other cell types is known and used within the tracheal community [20]. However the 2.7 kb DNA fragment used to prepare the *cut(ue)-*Gal4 line, which lies more than 80 kb upstream of the *cut* coding sequence, was not previously known to regulate expression in the larval airways.

That *cut(ue)-*Gal4 produces larval lethality when driving *lov* RNAi clearly indicates that *lov* is also expressed in the embryonic/larval tracheal system. Our prior analysis of embryonic Lov expression did not detect Lov protein in the developing tracheae [6], but formation of the impermeable cuticle prevents protein immunolocalization late in embryogenesis when the tracheae mature. As an alternative approach, we examined the early expression pattern for Gal4 produced by the *lov* PGawB enhancer trap [8] mutation *lov^91Y^*-Gal4. Previously, we investigated the Gal4 pattern from this insertion in the adult brain, in connection with its effects on adult gravitaxis behavior [5]. Using a UAS-mCherry reporter, no Gal4 expression was detected for *lov^91Y^*-Gal4 in early embryogenesis, but later in development, mCherry expression faithfully mirrored the expression pattern of Lov in the nervous system that we have characterized previously [6]. However, in addition, faint mCherry expression was detected in all the epithelial cells of the tracheae in the very final stages of embryogenesis. Fluorescence was strongest in the fusion cells whose role is to fuse the individual segmental sections of the tracheal DTs into continuous tubes (Fig 1A and 1D). The weak tracheal epithelial expression continued beyond hatching throughout the larval stages (Fig 1D).

We confirmed that this *lov^91Y^*-Gal4 expression in the tracheae is a faithful representation of *lov* gene activity by staining third instar larval tracheae with Lov antibody. Nuclear Lov staining was detectable in all the epithelial cells and fusion cells of the DTs (Fig 1E). Our prior studies showed that by the end of embryogenesis, expression of Lov in many neural cell types ceases. In particular only a small subset of neurons in the PNS continues to express Lov in the larval stages. We found that in *lov^91Y^*-Gal4, this subset of the neurons also expresses Gal4 in the larval period (data not shown). We conclude that *lov^91Y^*-Gal4 faithfully reports the *lov* expression pattern from late embryogenesis into larval life and reveals both neural and tracheal *lov* expression at these stages. The Lov tracheal expression pattern includes the regions of the tracheae in which *cut(ue)-*Gal4 is active. Thus the lethality of *cut(ue)*-Gal4 > *lov* RNAi larvae reflects previously unknown activity in the tracheae for both *lov* and the *cut(ue)*-Gal4 construct.

### A range of behavioral and developmental phenotypes for *lov* knockdown in the tracheae identified using a burrowing/tunneling assay

Initial observation of *cut(ue)*-Gal4 > *lov* RNAi larvae indicated two behavioral abnormalities: they stayed away from their food and did not tunnel into an agarose substratum during wandering. To further characterize and quantitate these behaviors and to confirm their origin in *lov* knockdown in the tracheae, we generated and used an assay that assesses larval food burrowing, substratum tunneling, and overall development (Material and Methods) and examined larvae in which several Gal4 lines expressing in the tracheal system were used to drive *lov* RNAi. Ultimately, detailed studies were performed for three drivers—*breathless* (*btl*)-Gal4, which is expressed throughout the tracheal system from early in its inception in embryogenesis [21], *cut(ue)-*Gal4 and *lov^91Y^*-Gal4. The PG142 Gal4 line was not used because this driver also gave Gal4 expression in the larval musculature. The expression patterns of the three chosen Gal4 lines in first instar larvae are compared in Fig 1. These patterns were imaged using identical exposure times so as to convey the relative strengths of Gal4 expression. Gal4 drivers for the developing posterior spiracles [22] produced no effects, confirming our finding that *lov^91Y^*-Gal4 > UAS mCherry larvae do not express mCherry in these spiracles (data not shown).

**Fig 2:**
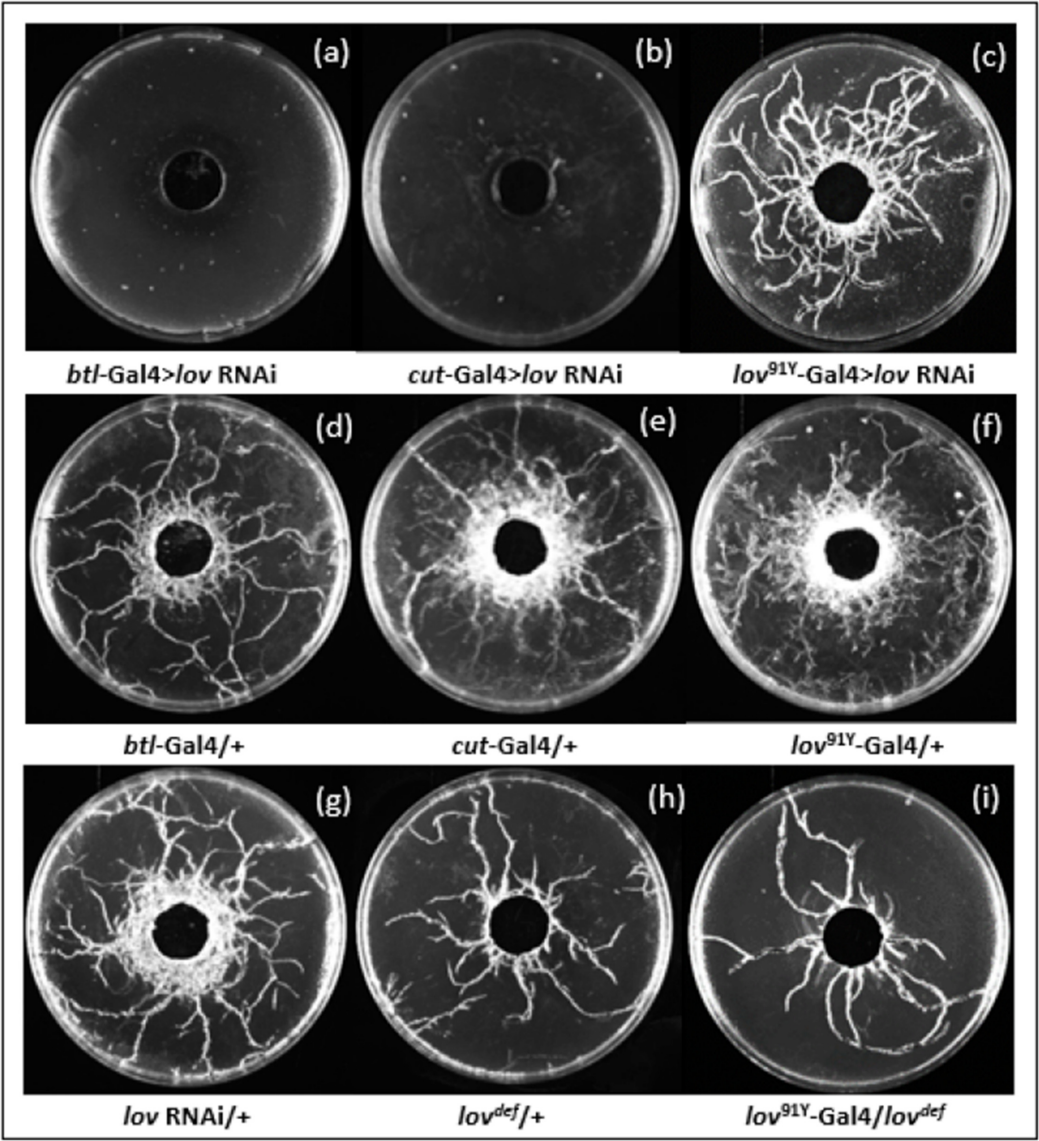
Tunneling behavior of various genotypes examined in these studies. Images of cleaned agarose plates from tunneling assays for key genotypes are shown. Tunneling was quantitated using Image J. Five control genotypes (panels (d)-(h)) show robust tunneling. *lov^91Y^*-Gal4 > RNAi larvae show comparable tunneling activity (panel (c)) but tunneling is completely absent for *cut(ue)-*Gal4 > RNAi and *btl*-Gal4 > RNAi larvae (panels (a) and (b). *lov^91Y^*-Gal4 /*lov def* larvae show limited tunneling behavior (panel (i)).

**Fig 3:**
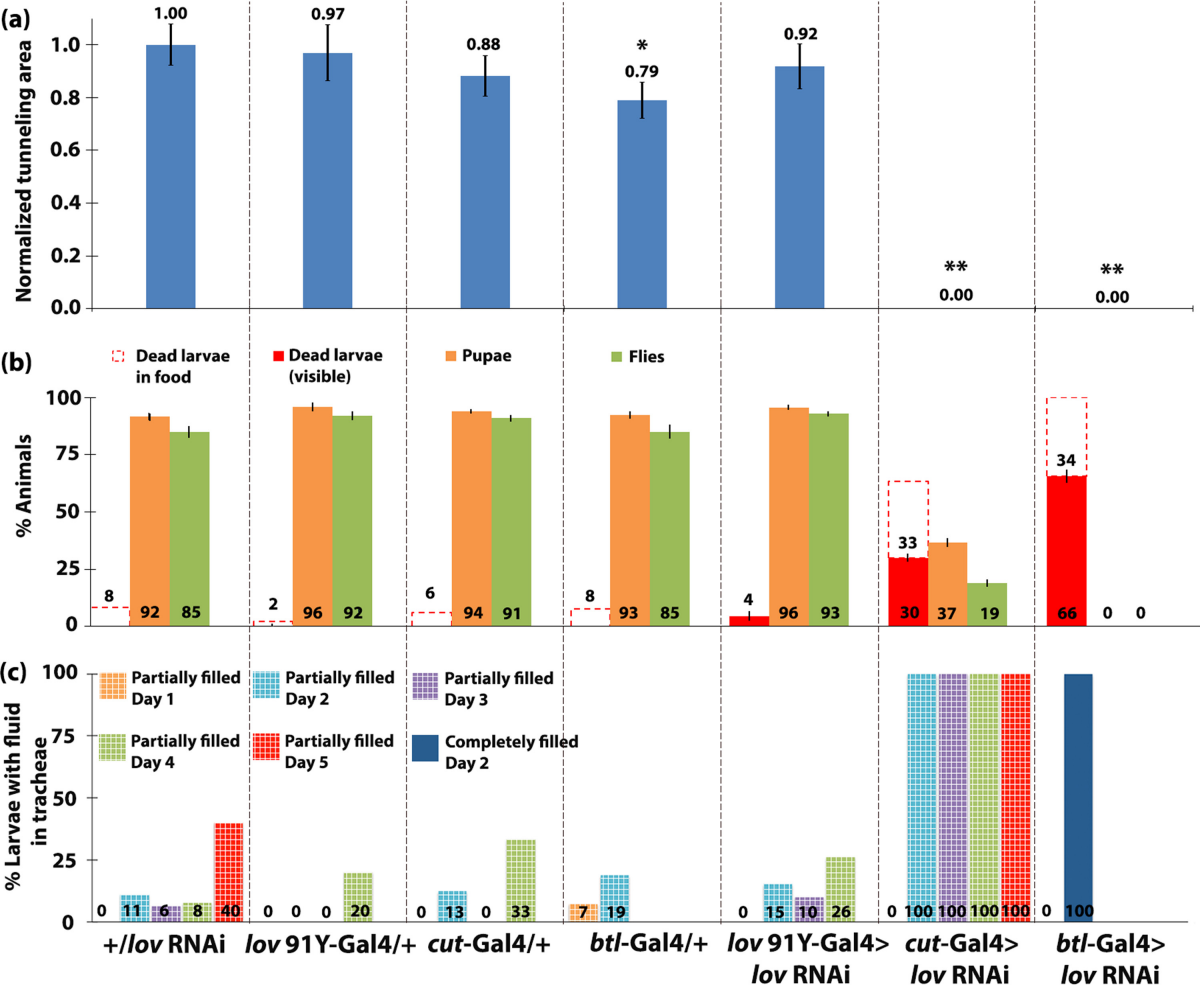
Failure to burrow, tunnel and survive to adulthood correlate strongly with fluid accumulation in the tracheae. (a) Quantitation of tunneling was performed as described in Material and Methods for the genotypes shown. Mean values ± SEMs for the tunneling areas were calculated and then normalized to the value for the +/*lov* RNAi control. P values, Student’s t-test, * = P< 0.01, ** = P<0.00001, compared to +/ *lov* RNAi control. b) For larvae subjected to the burrowing/tunneling assay, the average percentage that died in the food, or outside the food (an indication of failure to burrow) was determined, in addition to the average percentage that pupated and the average percentage that emerged as adults. (c) Larvae of the same genotypes as those used for burrowing/tunneling assays, were imaged daily and the images were used to assess accumulation of fluid in the DTs (see Material and Methods). The percentages of larvae with partially fluid-filled DTs (checkered bars) or completely fluid-filled DTs (solid bars) on days 0–5 post-hatching are shown.

*lov* tracheal knockdown with the three Gal4 lines produced effects of differing severity on larval growth and behavior. Fig 2 shows images of tunneling assay plates for the key genotypes and Fig 3 and S2 Fig show quantitation of food burrowing, substratum tunneling and survival to pupation and adulthood for these larvae and other relevant genotypes. For *cut(ue)*-Gal4 > *lov* RNAi, about half of the larvae were found away from their food in the first 2–3 days after hatching (S2 Fig) and they showed a dispersion in size as they grew, with some larvae almost normal in size and others noticeably smaller than controls. By day 5 after hatching ~30% of the larvae had died and a further ~30% died before pupation (Fig 3B). Strikingly, those that reached wandering phase showed no tunneling at all in the agarose substratum (Fig 2B). Only ~ 20% of the larvae survived to adulthood. The effects of *lov* knockdown with *btl*-Gal4 were even more detrimental. Larvae were sluggish and showed very little growth, with ~65% dying outside the food during days 1–5 after hatching (Fig 3B and S2 Fig). As for *cut(ue)*-Gal4, no larvae tunneled in the agarose substratum (Fig 2A), but in contrast to *cut(ue)*-Gal4, no larvae survived long enough to attempt pupation. For *lov^91Y^*-Gal4 > *lov* RNAi, essentially no defects in the parameters measured were detected: larvae grew and developed to adults like controls, burrowing in their food and tunneling during wandering in a wild type manner (Figs 2C,3A and 3B).

**Fig 4:**
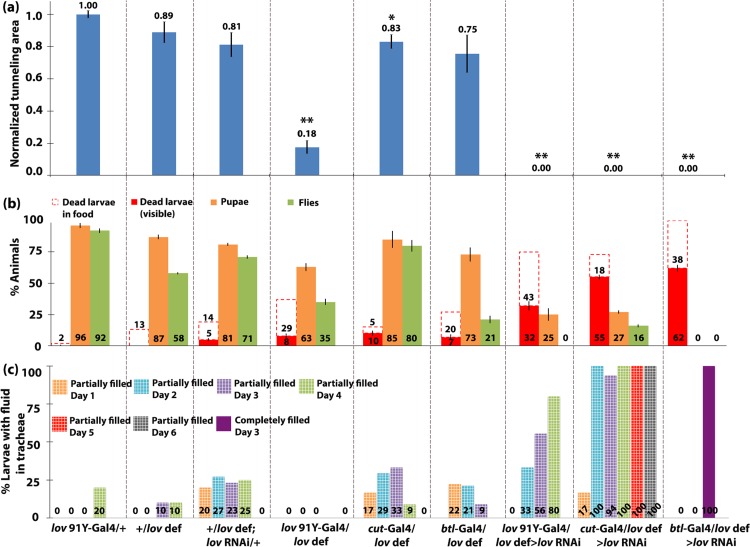
Correlation of failure to burrow, tunnel, and survive to adulthood, with tracheal fluid-filling in *lov* hemizygous genotypes. Data for the experimental and control genotypes as in Fig 3 in a *lov* hemizygous background. All data were generated and shown as in Fig 3, except that in (a) the quantitated tunneling areas are normalized to the value of *lov^91Y^*-Gal4 /+. P values, Student’s t-test, * = P< 0.01, ** = P<0.00001, compared to the *lov^91Y^* -Gal4 /+ control. Note i) in the hemizygous condition, *lov^91Y^* -Gal4 /*lov def* > *lov* RNAi larvae fail to tunnel and ii) *lov^91Y^* -Gal4 /*lov def* larvae show reduced tunneling despite having air-filled tracheae.

The phenotypic differences noted for the three Gal4 lines correlate well with their differences in strength and location of Gal4 expression. As shown in Fig 1, *cut(ue)*-Gal4 expression is very weak in most of the tracheal network, but is strong in the most posterior sections of the tracheal DTs, close to the spiracles. *btl*-Gal4 and *lov^91Y^*-Gal4 are expressed throughout the tracheal epithelial cells but *lov^91Y^*-Gal4 expression does not begin until the tracheal system is fully formed and is much weaker than *btl*-Gal4 expression (Fig 1). To address whether the behavioral and developmental differences seen between the lines result from differences in *lov* knockdown, all three lines were also tested in a *lov* hemizygous background (Fig 4).

With one copy of the genomic *lov* gene removed, *lov^91Y^*-Gal4/*lov def* > *lov* RNAi larvae showed behavioral/development effects similar to those seen with the other tracheal Gal4 drivers in a wild type background, including a complete failure to tunnel and considerable larval death away from the food (Fig 4 and S2 Fig). The *cut(ue)*-Gal4 > *lov* RNAi phenotype was also enhanced in *lov* hemizygotes, resulting in growth, behavior and viability defects more like those seen with *btl*-Gal4 (Fig 4 and S2 Fig) in a wild type *lov* background. The phenotype of *btl*-Gal4/ *lov def* > *lov* RNAi was not significantly different to that of *btl*-Gal4 > *lov* RNAi. These findings indicate a strong correlation between extent of *lov* knockdown in the tracheae and the behavioral and developmental effects seen.

### Strong correlations between growth, behavior and tracheal damage

We examined the state of the larval tracheae for the various genotypes described above. Normally, air present in the tracheae causes light refraction and the outlines of the tracheal walls are highly visible as black lines through the larval body wall. In contrast when the tracheae are fluid-filled, the tracheae are almost invisible against the hemolymph-filled larval interior. The tracheae contain fluid at the end of embryogenesis but very shortly before hatching, they are inflated by a fluid removal process thought to involve the *pickpocket* class of ion channels [23]. Notably, in all larvae expressing *lov* RNAi in the tracheae, including the most strongly affected genotype, *btl*-Gal4/*lov def* > *lov* RNAi, the tracheae were water-tight and air-filled immediately after hatching. This finding indicates that *lov* plays no role in the initial inflation of the tracheae.

**Fig 5:**
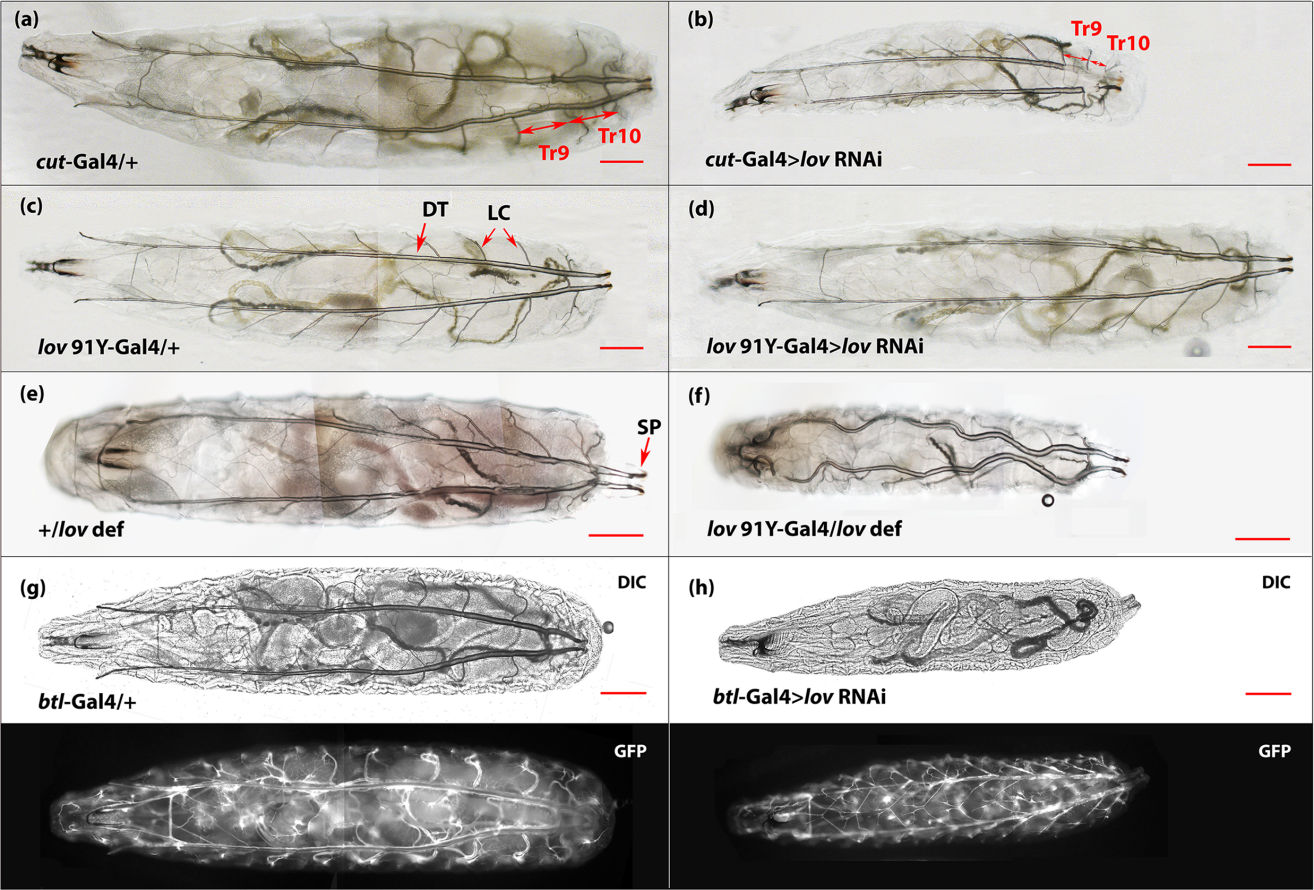
Fluid-filling of the DTs in larvae of the genotypes studied. Bright field images of dorsal views of control (left-hand column) and experimental (right-hand column) second instar larvae for the three Gal4 > *lov* RNAi genotypes studied and for *lov^91Y^* -Gal4 in the hemizygous condition. The *btl*-Gal4 construct used has UAS-*actin*-GFP sequences on the same chromosome. This allowed GFP imaging of the tracheal system (shown in the (g) and (h) lower panels). Air in the tracheae produces light refraction making the outlines of the DTs and lateral connective tracheal branches visible in control larvae. Fluid in the tracheae renders the tracheae almost invisible. For *btl*-Gal4 > *lov* RNAi (h), although grossly morphologically normal (compare (g) and (h) lower panels) the entire tracheal system is fluid-filled, whereas for *lov^91Y^* -Gal4 > *lov* RNAi (d), the entire system is air-filled. In *cut(ue)-*Gal4 > *lov* RNAi larvae (b), region Tr10 of the DTs is always fluid-filled and is noticeably shorter. In this larva, Tr9 is also fluid-filled in one of the DTs. *lov^91Y^* -Gal4 /*lov def* larvae (f) show a different phenotype: the tracheae are air-filled but overall larval growth is inhibited producing convoluted tracheae in some larvae. LC = lateral connectives, SP = spiracles. Scale bars = 200 μm.

However, beyond hatching, fluid accumulation in the tracheae was seen amongst the genotypes studied. Figs 3 and 4 present quantitation of the tracheal fluid-filling for all genotypes examined and Fig 5 shows tracheal images for larvae of the key genotypes studied. The extent of larval tracheal fluid-filling correlated strongly with the growth and behavioral phenotypes described above. Negligible fluid accumulation was seen in genotypes that grew, burrowed, and tunneled normally, including *lov^91Y^*-Gal4 > *lov* RNAi. In contrast, all but one genotype (see below) that showed poor growth, failed burrowing and tunneling, and high pre-adult death showed strong penetrance of the tracheal fluid-filling phenotype. For *btl*-Gal4 > *lov* RNAi and *btl*-Gal4/*lov def* > *lov* RNAi larvae, which show the most severe developmental effects, the entire tracheal system becomes fluid-filled as early as day 2 after hatching in 100% of the larvae (Fig 5). In *cut(ue)*-Gal4 > *lov* RNAi larvae, although the fluid-filling phenotype is 100% penetrant, the expressivity is more limited than in *btl*-Gal4 > *lov* RNAi larvae (Figs 3–5). Typically only short regions of the DTs, corresponding to the regions showing high Gal4 expression under the *cut(ue)-*Gal4 driver (Fig 1) are fluid-filled. This more limited tracheal damage probably underlies the ability of some of these larvae to reach wandering stage and express the failed tunneling phenotype.

The larvae that proved exceptional to this correlation of behavioral/developmental defects with tracheal fluid accumulation were *lov^91Y^*-Gal4/*lov def* larvae. In addition to being a *lov* Gal4 enhancer trap insertion, *lov^91Y^* is a *lov* mutation in its own right, initially identified for the defective gravity responses it produces [6]. Further analysis of the *lov^91Y^*-Gal4/*lov def* larval phenotype is discussed below.

### Are the tracheal *lov* knockdown behavioral defects a consequence of hypoxia?

**Fig 6:**
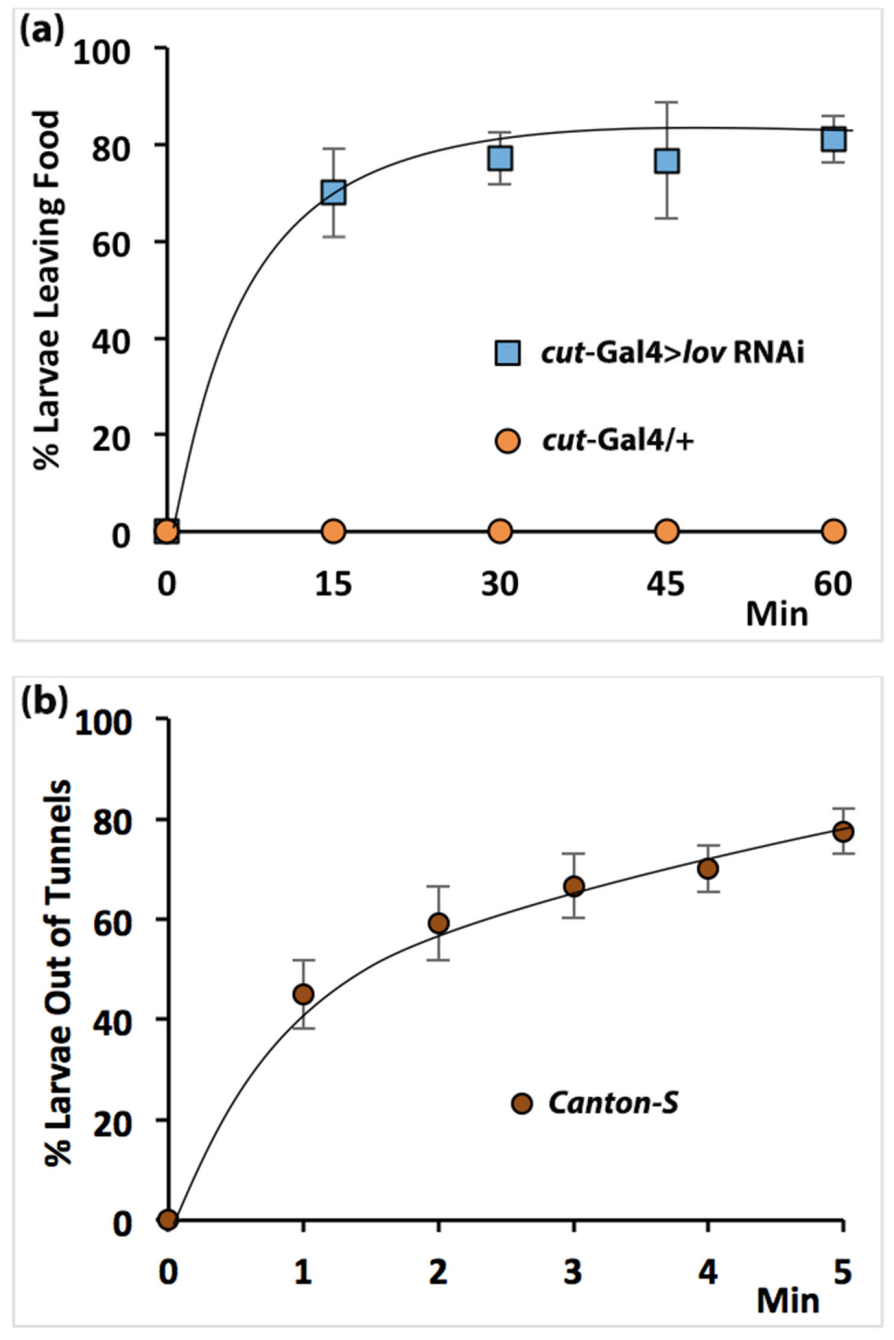
Failure to burrow and tunnel mimic hypoxia-induced behaviors. (a) *cut(ue)****-***Gal4 > *lov* RNAi larvae or control larvae were buried under a pile of yeast paste and the number of larvae that crawled out of the pile was monitored over time. Control larvae stayed in the yeast but the experimental larvae left the paste in numbers and at a rate comparable to findings for hypoxic larvae [13], n = 200 in batches of 10 larvae. Error bars = +/- SEM. (b) Wandering wild-type Canton-S larvae, tunneling in plates as used for the tunneling assay, were subjected to an atmosphere of N2 gas and the number of larvae that left their tunnels and moved to the agarose surface was monitored over time. N = 120 in batches of 10 larvae. Error bars = +/-SEM.

Fluid-filling of the tracheae will inevitably limit oxygen exchange in the larva suggesting that both the behavioral and growth effects of *lov* tracheal knockdown reflect responses to hypoxia. The aberrant behaviors of *lov* tracheal knockdown larvae reported here are strikingly similar to a larval response to hypoxia described previously. Normal larvae feeding on piles of yeast paste burrow into their food headfirst, but keep their posterior spiracles outside the food, exposed to the air. Wingrove and O’Farrell [13] established that in hypoxic conditions, third instar larvae abandon this feeding position and rapidly move out of their food. To establish whether third instar *cut(ue)*-Gal4 > *lov* RNAi larvae will leave the interior of a yeast pile like hypoxic larvae, we placed them under mounds of yeast paste and monitored their behavior as compared to controls. As shown in Fig 6, whereas control (*cut(ue)*-Gal4 /+) larvae stayed in the food, *cut(ue)*-Gal4 > *lov* RNAi larvae came out of the yeast pile in a manner comparable to that of wild type larvae subjected to 1% oxygen (Fig 6A—compare Fig 1F, Wingrove and O’Farrell 332 [13]).

To determine whether failure to tunnel is also a response to hypoxia, we subjected wild type larvae that were actively tunneling in the agarose substratum to very low oxygen levels by flushing the Petri plates with nitrogen gas. As shown in Fig 6B, ~80% of the larvae quickly moved out of their tunnels onto the agarose surface under these conditions. Thus in wild type larvae, absence of tunneling is a behavioral response to lack of oxygen.

In wild type larvae, these hypoxia-induced behaviors are rapidly initiated by sensory neurons some of which contain atypical soluble guanylyl cyclases that act as oxygen sensors (reviewed in [24]). However, interpreting the behavioral defects of *cut(ue)*-Gal4 > *lov* RNAi larvae as purely neuronal responses to hypoxia is complicated by the recognition that long- lasting hypoxia depresses metabolism and produces both growth and behavioral defects [25, 26]. *cut(ue)*-Gal4 > *lov* RNAi larvae that survive to wandering stage and fail to tunnel are noticeably smaller than controls (Fig 5B) and are sluggish. *btl*-Gal4 > *lov* RNAi larvae are even more strongly affected: they show very little growth (Fig 5H) and at death are comparable to larvae exposed to 3.5% oxygen during days two and three after hatching (see Fig 1, Reference [26]). Thus, an alternative explanation for failed burrowing and tunneling in *cut(ue)*-Gal4>*lov* RNAi is that these defects are indirect responses to prolonged hypoxia and reflect the fact that the larvae are too physiologically challenged to burrow and tunnel.

**Fig 7:**
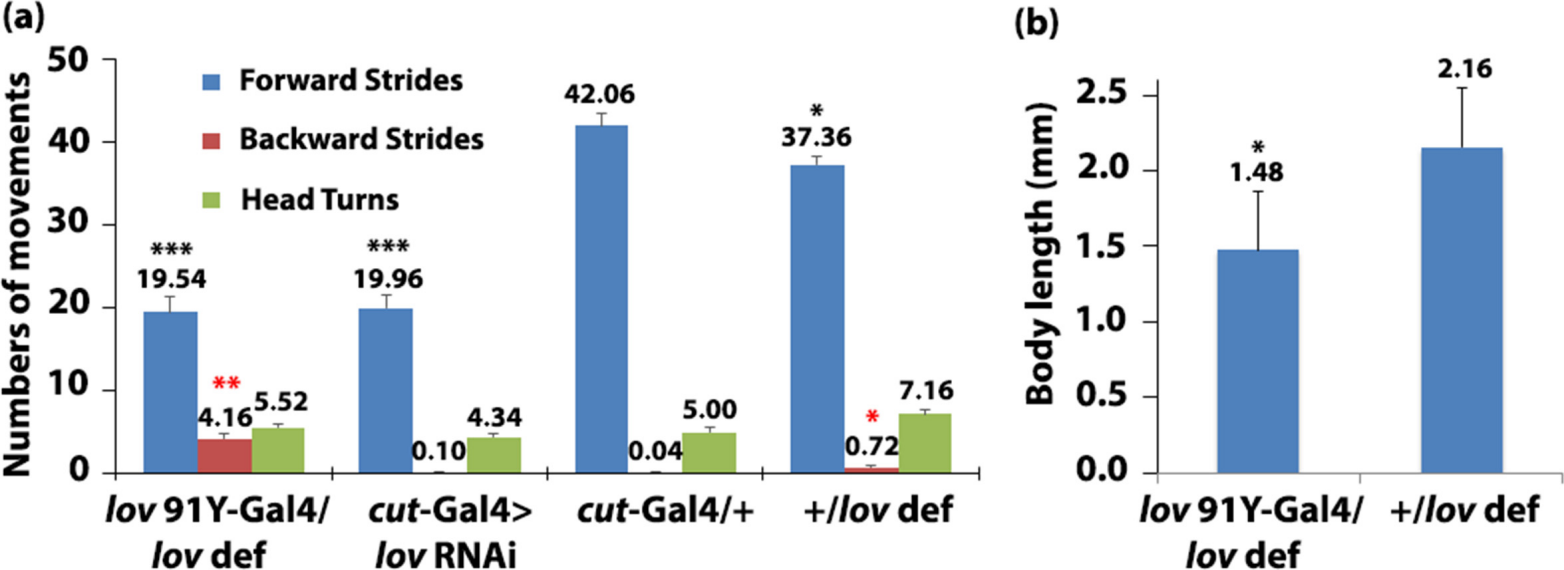
Locomotor defects of *lov^91Y^*-Gal4/*lov def* larvae compared to *cut(ue)*-Gal4>*lov* RNAi larvae and *lov^91Y^*-Gal4/*lov def* larval growth defects. (a) Larval locomotion was analyzed as described in Material and Methods, for forward and backward strides, and head turns. N = >50 for all genotypes. P values, Student’s *t*-test, black *** = P 377 <0.0001, black * = P < 0.01, red ** = P < 0.001, red * = P < 0.01, all compared to *cut*-Gal4/+. *lov^91Y^* -Gal4/*lov def* larvae are more compromised than *cut(ue)*-Gal4 > *lov* RNAi larvae. (b) Larval length measurements. At least 20 larvae for each genotype were imaged as described in Material and Methods. The images were processed with Adobe Photoshop and NIH Image J to measure the body length. P value, Student’s *t*-test, * = P <0.001 compared to +/*lov def*.

A comparison of the locomotor activity of *lov^91Y^*-Gal4 /*lov def* larvae with that of *cut*-Gal4 > *lov* RNAi larvae provides insight on this point (Fig 7). In the hemizygous condition, *lov^91Y^*-Gal4 produces effects on growth and survival to adulthood comparable to those seen for *cut(ue)*-Gal4 > *lov* RNAi (compare in Figs 3B and 4B and Fig 5B with 5F). But the tracheae remain open and air-filled in *lov^91Y^*-Gal4 /*lov def* larvae (Fig 5F) so they are unlikely to be hypoxic. Thus, in contrast to the *cut(ue)*-Gal4 > *lov* RNAi larvae, the mutant phenotypes of these larvae probably result from neural defects caused by the *lov^91Y^* insertion (see Discussion) rather than from loss of *lov* expression in the tracheae. Upon testing, *lov^91Y^*-Gal4 /*lov def* larvae proved more compromised in terms of locomotion than *cut(ue)*-Gal4 > *lov* RNAi larvae: although forward locomotion rates are comparable and low for the two genotypes, *lov^91Y^*-Gal4 hemizygous larvae are also hampered by spontaneous backward motion (Fig 7A). Nevertheless *lov^91Y^*-Gal4 /*lov def* larvae burrow into their food (S2 Fig) and show tunneling (Fig 2I), quantitated as ~18% that of the averaged control value (Fig 5A), whereas ~50% of *cut(ue)*-Gal4 > *lov* RNAi larvae do not burrow (S2 Fig) and their failure to tunnel is absolute (Fig 3A). This comparison suggests that the burrowing /tunneling defects in *cut(ue)*-Gal4 > *lov* RNAi are not due to indirect effects on growth and locomotion but rather are a direct neural response to hypoxia.

To identify direct evidence of hypoxia in *lov* tracheal knockdown larvae we examined the expression of genes known to be induced by low oxygen in larval life. Responses to hypoxia show strong developmental regulation in Drosophila. In particular, late embryos and early larvae show very limited changes in gene expression in hypoxia, with no increase in transcripts for the canonical indicator of hypoxia—lactate dehydrogenase (LDH) [27]. This probably reflects the fact that embryos and early larvae rely heavily on **aerobic** glycolysis (lactate production in the presence of oxygen), with concomitant upregulation of LDH [28]. In contrast, wandering third instar larvae show significant transcriptional responses to hypoxia, with LDH showing the greatest upregulation of all genes examined [27]. Given that *btl*-Gal4 > *lov RNAi* larvae die before this stage, we thus focused on determining whether wandering *cut(ue)-*Gal4 > *lov* RNAi larvae/early prepupae show hypoxia-induced transcriptional changes.

**Fig 8:**
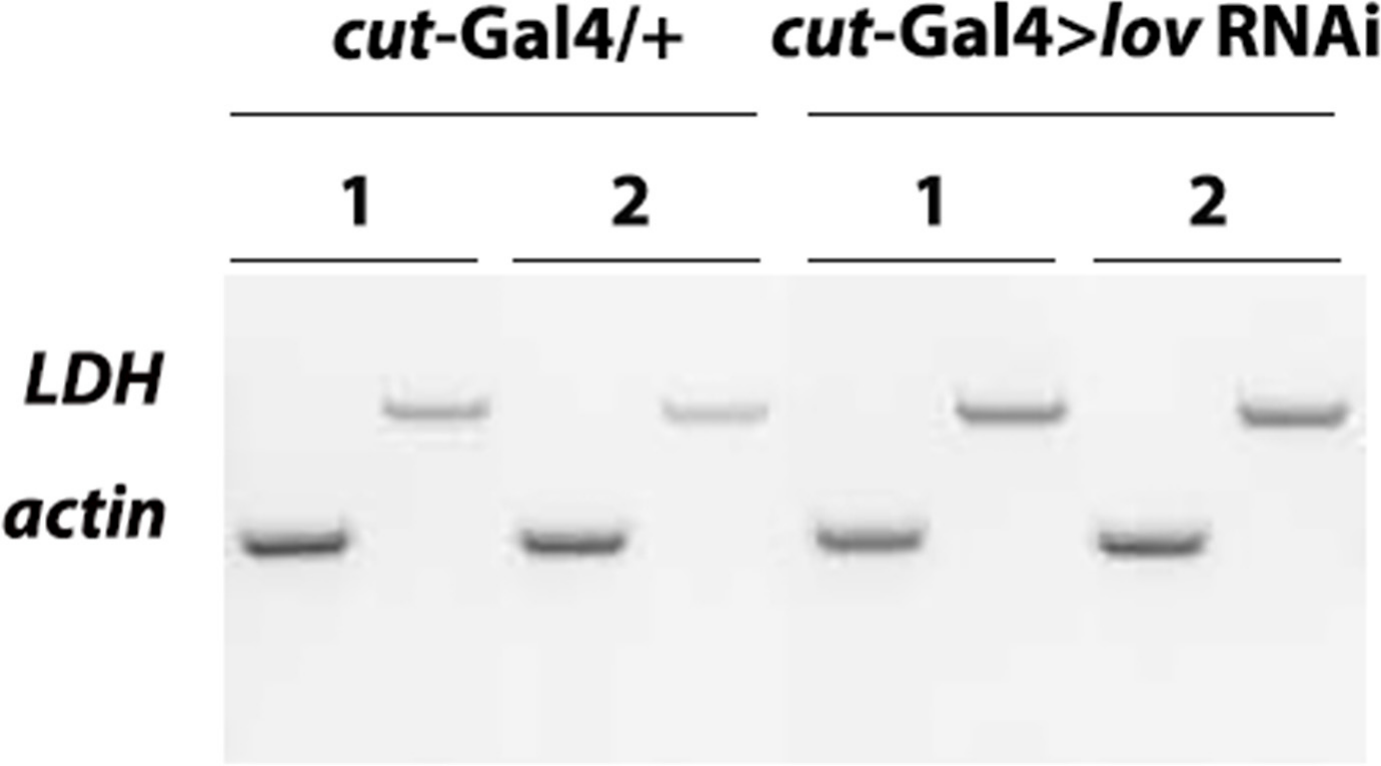
LDH expression is up-regulated in *cut(ue)*-Gal4 > *lov* RNAi larvae. Late wandering third instar larvae were collected for RNA extraction. Semi-Q RT-PCR was used for transcript analysis. *LDH* and *actin* PCR products for each RNA sample were run in parallel in separate gel lanes. 1 and 2 indicate RT-PCR products from two independent RNA preparations. Four RNA preparations total gave comparable results. For quantitation of LDH upregulation in *cut(ue)-*Gal4 > *lov* RNAi larvae, band intensities for the LDH and actin PCR products were quantitated with NIH image J and used to calculate LDH/actin (L/a) ratios for control and experimental (*cut(ue)-*Gal4 > *lov* RNAi) samples. The fold increase in LDH expression in the experimental samples was then calculated as the average value for L/a _experimental_/L/a _control_.

We used Semi-Q RT-PCR to probe for transcripts from the four genes shown previously to undergo the greatest hypoxia-induced upregulation at this developmental stage [27]; these are *CG10160*, the gene for LDH (also known as ImpL3 in Drosophila); *CG11652*, the gene for *dDPH1*, a component of diphthamide synthesis; *CG4608*, the gene for the FGF receptor, Branchless, and *CG31543*, the gene for Fatiga, the prolyl hydroxylase that regulates levels of the key hypoxia-induced transcription factor HIF-α (Sima in Drosophila) [27]. No consistent increase in expression for *fatiga* and dDPH1 was detected, with *branchless* transcripts showing a minor but consistent upregulation relative to control (data not shown). In contrast, transcripts for LDH were consistently upregulated about two fold (2.3 +/- 0.5) in *cut(ue)*-Gal4 > *lov* RNAi larvae as compared to controls (Fig 8). Given the key role of this enzyme in anaerobic respiration, we conclude that *cut(ue)*-Gal4 > *lov* RNAi larvae are hypoxic.

### The *lov^91Y^*-Gal4 /*lov def* phenotype

We found that *lov^91Y^*-Gal4 hemizygous larvae are phenotypically distinct from larvae with tracheal knockdown of *lov*, both in terms of behavior and morphology. As discussed above, behaviorally they differ in that they display spontaneous backward locomotion and some tunneling activity and morphologically they have air-filled tracheae. In addition, although, like tracheal *lov* RNAi larvae, they are smaller than controls (Fig 7B), their reduced size shows evidence of a disconnect between the growth of the tracheae and the body at large. Thus, 50% of the *lov^91Y^*-Gal4 hemizygous larvae examined had highly convoluted tracheae indicating tracheal growth in excess of overall growth (Fig 5F). In contrast, mild tracheal convolution was seen in only 14% of the control larvae (data not shown). Several aspects of the *lov^91Y^*-Gal4 hemizygous phenotype are similar to those detected previously for mutation *lov^47^* [6]. The mutation deletes DNA close to the PGawB insertion site in *lov^91Y^* resulting in loss of Lov expression in many neural phenotypes. We address these similarities in the Discussion.

### *lov* knockdown in the tracheae inhibits growth and polyploidization of the tracheal epithelial cells in larval life

We found only minor structural defects in the late embryonic/early larval tracheal system of *btl-*Gal4 > *lov* RNAi larvae (data not shown). Further, the tracheae in *btl-*Gal4 > *lov* RNAi larvae inflate normally just before hatching. Thus the tracheal leakiness detected here appears to reflect a role for *lov* during tracheal growth and development in larval life.

One cause of larval tracheal fluid-filling is loss of the lipid water barrier within the posterior spiracles. A simple assay in which a dye is added to semi-liquid food makes spiracle water entry easily detectable [29]. Using this assay we saw no dye within the spiracles in *cut(ue)*-Gal4 > *lov* RNAi larvae (data not shown), even though there are fluid-filled tracheal regions very close to the spiracles in these larvae.

**Fig 9:**
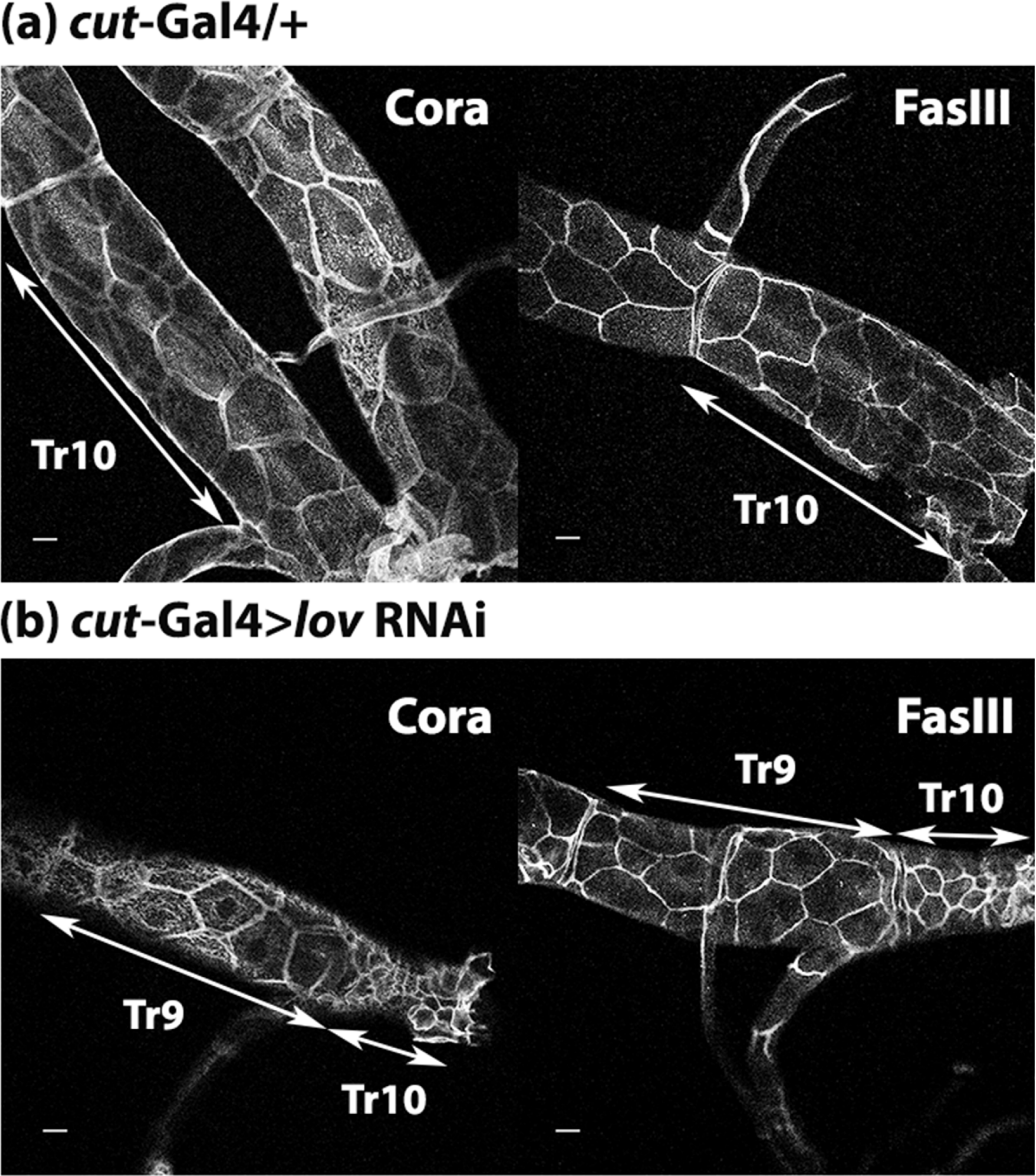
*cut(ue)*-Gal4 > *lov* RNAi larval tracheae show normal immunostaining for septate junction constituents. Confocal images of (a) *cut(ue)*-Gal4/+ and (b) *cut(ue)*-Gal4 > *lov* RNAi tracheae stained with either Coracle (Cora) or Fas III antibodies. Tracheae were dissected from third instar larvae as described in Material and Methods. Staining at the plasma membranes for both proteins is similar in control and *lov* knockdown tracheae but the cells of Tr10 are much smaller in the *cut(ue)*Gal4 > *lov* RNAi genotype. Scale bars = 20 μm.

To look for defects in the tracheae proper, we took advantage of the extremely regional expression of Gal4 seen with the *cut(ue)-*Gal4 driver. Under this driver, adjacent sections of the tracheal trunks showing weak (Tr9) or strong (Tr10) *lov* RNAi knockdown can be compared. We therefore developed a protocol to dissect out the dorsal trunks from late stage larvae to examine these tracheal regions in *cut(ue)-*Gal4 > *lov* RNAi and control animals. Given that the septate junctions (equivalent to vertebrate tight junctions) provide the barrier to fluid passage through the tracheal epithelial cell layer [30], we first investigated the expression of two key septate junction proteins (Coracle (Cora), and Fas III). These proteins were both present at high levels on the cell membranes of Tr9 and Tr10 in both *cut(ue)*-Gal4 > *lov* RNAi and control larvae. At the light microscope level, no differences in localization of these proteins between control and experimental tissues was detected (Fig 9).

However, in performing these immunolocalization studies, an unexpected finding emerged. As shown in Fig 1B and 1G, the *cut(ue)-*Gal4 driver produces a very specific pattern of Gal4 expression in the DTs, with low levels in Tr9, high levels in Tr10, and even higher levels in the bridge and the extreme terminal sections of the DTs adjacent to the spiracles. We found that, in *cut(ue)-*Gal4 > *lov* RNAi, the size of the DT epithelial cells decreases dramatically in the region of high Gal4 expression. Tr9 cells appear somewhat smaller than in control larvae and Tr10 cells are considerably reduced compared to Tr9 cells (Fig 9). In wild type larvae, cells posterior to the bridge are already noticeably smaller than in the rest of the DTs (data not shown) and in the *cut(ue)-Gal4* > *lov* RNAi larvae these cells were so small and disorganized that they were usually no longer attached to the spiracles. A break in the continuity of the DTs that would allow hemolymph entry was therefore present at this position.

**Fig 10:**
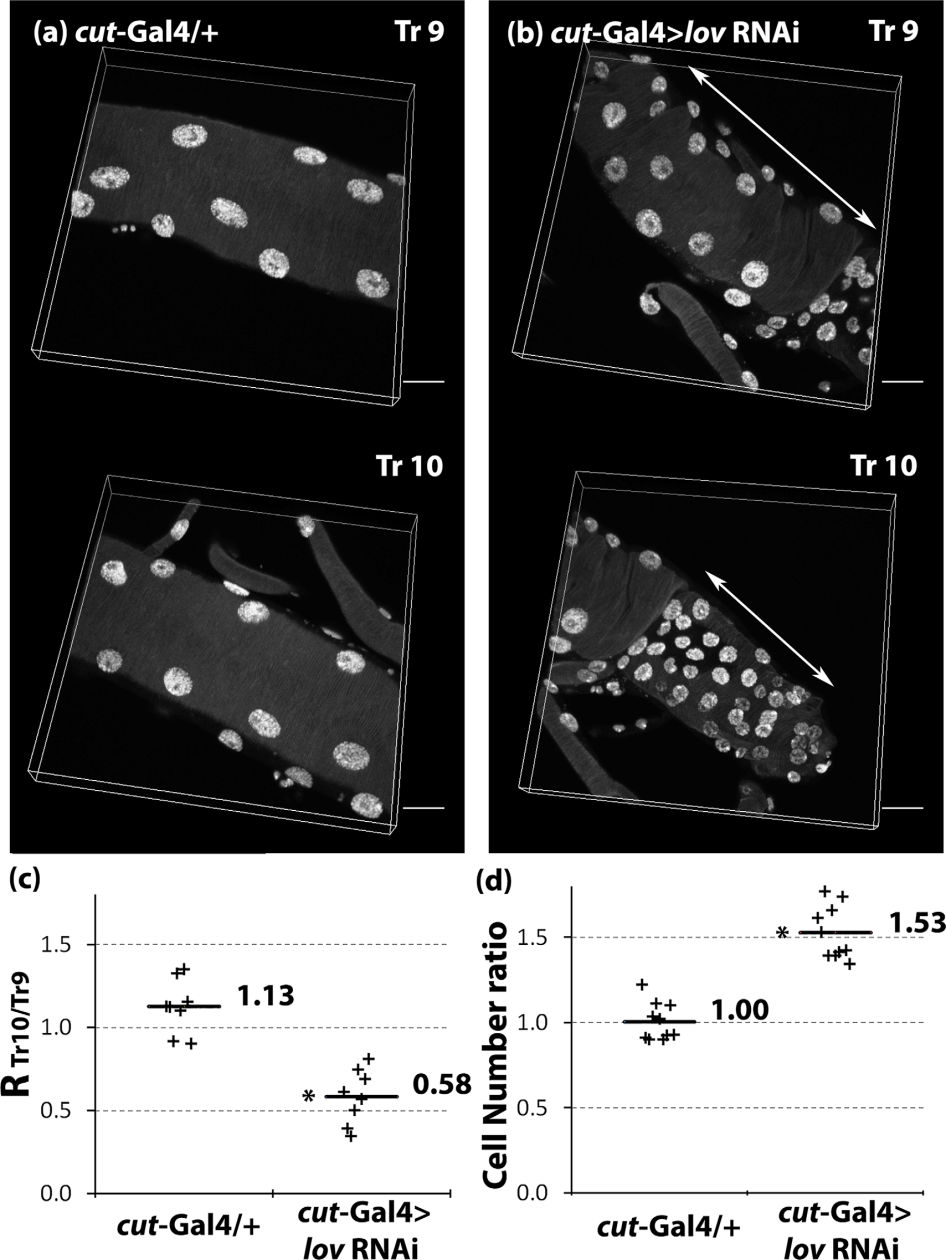
In *cut(ue)*-Gal4 > *lov* RNAi tracheae, cells of DT Tr10 are smaller, more numerous, and show decreased DNA content compared to adjacent cells in DT Tr9. DAPI stained nuclei in confocal sections of DT metameres Tr9 and Tr10 from (a) *cut(ue)*-Gal4 /+ and (b) *cut(ue)*-Gal4 > *lov* RNAi third instar tracheae. (c) After DAPI quantitation (see Material and Methods) an RTr10/Tr9 value was calculated for all control or *lov* knockdown tracheae examined. RTr10/Tr9 = average of DAPI intensity per nucleus (Tr10)/average of DAPI intensity per nucleus (Tr9)). Horizontal bars show average RTr10/Tr9 values. The average RTr10/Tr9 value for *cut(ue)*-Gal4 > *lov* RNAi is approximately half that of *cut(ue)-*Gal4 /+ larvae. (d) The cell number ratios for control and *lov* knockdown tracheae in Tr9 and Tr10. Cell number ratios = cell numbers in Tr10/cell numbers in Tr9 and were calculated as described in Material and Methods. An increase in cell number of ~50% is indicated in Tr10 compared to Tr9 for *cut(ue)-*Gal4 > *lov* RNAi, as compared to control. Each + represents data collected from an individual larva. Scale bars = 20 μm. P value, Student’s *t*-test, * = P <0.00002 for *cut(ue)*-Gal4 > l*ov* RNAi compared to *cut(ue)*-Gal4 /+.

Although the cells in Tr10 were strongly affected by the *lov* knockdown, in most dissected DTs the Tr10 metamere was sufficiently intact to allow further analysis. DAPI nuclear staining revealed that the nuclei in Tr10 cells were noticeably smaller than those in Tr9 and there appeared to be more cells in this region than in the equivalent control region (Fig 10). Quantitation of the average ratio of Tr10/Tr9 nuclear fluorescence for cells in *cut(ue)-*Gal4 > *lov* RNAi and *cut(ue)-*Gal4 /+ larvae established that, whereas in controls, the nuclear DNA content of cells in Tr9 and Tr10 is similar, in *lov* knockdown larvae the DNA content of Tr10 cells is about half that of Tr9 cells (Fig 10). We also calculated the average Tr10/Tr9 ratio of nuclear numbers for control and *cut(ue)-*Gal4 > *lov* RNAi tracheae as an indication of the number of cells in each segment. Whereas the cell numbers for Tr9 and Tr10 are comparable in controls, in the *lov* knockdown tracheae, we quantitated a 50% increase in cell number for Tr10 over Tr9 (Fig 10). This quantitation probably underestimates the increase in cell number in Tr10. In *lov* knockdown DTs, the region posterior to Tr10 containing the smallest cells is almost always damaged such that its boundary with Tr10 is obscured. We therefore delineated the region we called Tr10 conservatively and thus probably excluded some Tr10 cells from our calculations.

## Discussion

### The link between *lov* tracheal expression and burrowing and tunneling behaviors

The failed food burrowing and substratum tunneling identified by this work led us to discover a new role for *lov* in the post-embryonic growth of the tracheal system. Although our expectation was that these behaviors originated in neural functions for *lov*, we found that they arise indirectly from a breach in tracheal integrity induced upon lowering Lov levels in the tracheae. This breach leads to tracheal fluid-filling, hypoxia, and finally, hypoxia-activated behaviors. These hypoxia-activated behaviors cannot originate in Lov-expressing neurons since the key tracheal driver used here, *cut(ue)-*Gal4, does not express in any neural elements. Rather, we hypothesize that the guanylyl cyclase-expressing neurons identified by the Morton laboratory as oxygen-sensing neurons [31] are the sensory elements that trigger the hypoxia-induced behaviors we have characterized here. Surprisingly, the role of *lov* that is disrupted to initiate these behaviors involves growth and polyploidization of the tracheal epithelial cells.

How might this decreased growth lead to loss of the barrier properties of the tracheal cells? As part of the 200-fold increase in weight during larval life [32], the larva body increases eight-fold in length [33]. Thus, suppression of tracheal cell growth will stretch the tracheae, a mechanical stress that could lead to physical rupture, allowing fluid entry. Our observations of the tracheal regions showing *lov* knockdown under the *cut(ue)*-Gal4 driver support this hypothesis. The DTs were frequently broken in the region posterior to Tr10 where the *lov* knockdown levels were highest. This region, and the adjacent Tr10, were always fluid-filled in the *lov* knockdown larvae.

This explanation for the origin of the tracheal damage assumes that loss of growth and endopolyploidy induced by *lov* knockdown precedes the tracheal damage and fluid-filling also produced by *lov* RNAi. As an alternative it could be argued that an unidentified initial defect causes tracheal leakage and then the presence of fluid in the tracheae inhibits tracheal growth. For example, despite our findings from light microscopy, we could propose that the septate junctions are not functional in *lov* knockdown, leading to fluid entry and subsequent growth inhibition. Preliminary experiments (data not shown), in which weaker *lov* knockdown in the tracheae was achieved by countering *cut(ue)-*induced Gal4 activity with leaky Gal80 expression, make this possibility unlikely. In these *cut(ue)-*Gal4/Gal80 > *lov* RNAi larvae, Tr10 growth is inhibited but there is no fluid accumulation in the tracheae.

### Systemic hypoxia in *cut(ue)*-Gal4 > *lov* RNAi larvae is limited

Our data indicate that the amount of tissue hypoxia in *cut(ue)-*Gal4>*lov* RNAi larvae is low when compared to that detected in comparable stage larvae exposed to 4% oxygen for six hours [27]. Although we found a consistent detectable increase in LDH gene expression, this increase is small compared the 14-fold upregulation of LDH RNA reported for systemic exposure to 4% oxygen. For three further genes that show substantial upregulation in 4% oxygen (*dDPH*, 11x increase, *fatiga*, 8x increase, *branchless*, 11x increase) we found small, in some cases inconsistent, changes in our *cut(ue)*-Gal4 > *lov* RNAi larvae.

Given the highly regional fluid-filling of the tracheae produced with the *cut(ue)* driver, we interpret this finding to mean that tissue hypoxia is mostly limited to regions surrounding the affected tracheal sections, that is, Tr9, Tr10 and the bridge region. The Morton laboratory has shown that the terminal sensory cones located on abdominal segments A8 and A9 contain neurons expressing oxygen-sensing guanylyl cyclases and are major oxygen sensing organs for the larva [24, 31, 34]. These sense organs are very close to the affected tracheal segments in *cut(ue)*-Gal4 > *lov* RNAi larvae and we propose they undergo sufficient oxygen deprivation in these mutant animals to activate the behavioral responses characterized here.

### The effects of *lov* on the growth and polyploidization of the tracheae

The morphogenesis of the Drosophila larval tracheal system during embryogenesis requires no growth by mitosis and cell division (for reviews see [35–37]). Instead, individual sections of the tracheae arise from clusters of epidermal cells that migrate inwards and undergo remarkable shape changes to produce a series of repeating, complex, branched structures in the segments of the developing embryo. Late in embryogenesis, so-called fusion cells at the tips of particular branches of each structure fuse these individual elements to give rise to the final continuous tubular system.

Subsequently, as for most of the larval tissues, the large increase in size of the tracheal system in larval life is accomplished by endoreplicative growth, that is, multiple rounds of DNA replication without nuclear or cytoplasmic division. This growth produces large cells each containing a single large polyploid nucleus. The precise point at which this endoreplicative growth is initiated is not known, but previous work indicates that most of the larval tissues have undergone at least one round of endoreplication prior to larval hatching [38].

Our examination of DT cells that have experienced *lov* knockdown since late in embryogenesis reveals that these cells are smaller, more numerous and reduced in DNA content. Comparisons to nuclei/cells in the imaginal discs, which remain diploid in larval life, make it clear however that the *lov* knockdown DT cells have undergone some endoreplicative growth. These findings raise the possibility that *lov* has a role in the initial commitment to endoreplication at the very end of embryogenesis, when *lov* expression in the DTs is initiated, and that when *lov* is depleted, this transition is delayed and instead some growth by mitotic divisions occurs. As discussed below, the cells in Tr2 of the DTs only grow by regular cell divisions, demonstrating that mitotic growth can occur in the larval DTs.

Our quantitation was based on comparing nuclear parameters for Tr9 to those of Tr10 within the same DT. This was done to avoid introducing additional variation by comparing Tr10 regions from control and experimental animals. Although this quantitation defines the aspects of tracheal cell growth that are affected by *lov* knockdown, it is likely that it underestimates the effects of a complete loss of *lov* function on these parameters. One factor causing an under-estimation of cell number increase (conservative definition of the posterior Tr10 boundary) is described above. Other factors that probably lessen the effects detected include the activity of the *cut(ue)* enhancer in Tr9 as well as in Tr10 (see Fig 1) and incomplete *lov* knockdown by the RNAi construct used. Complete loss of *lov* activity in these cells might thus produce a population that fails to initiate endoreplicative growth entirely.

The molecular mechanisms regulating endopolyploidy within the tracheal system have not been investigated. However, in other endopolyploid tissues, the Insulin pathway has been identified as coordinating endopolyploidy with the nutritional status of the larvae [39] and the Notch pathway has been shown to coordinate endopolyploidy with tissue morphogenesis in the ovarian follicles [40].

### The *cut(ue)* enhancer and regional specialization of the tracheae

The Gal4 expression generated by the *cut(ue)* enhancer is strikingly limited, immediately suggesting a role for *cut* in regional specialization of individual DT metameres. It is noteworthy that the region of highest activity of this enhancer is the region closest to the spiracles, posterior to the “bridge” structure, where polyploidization of the tracheal epithelial cells is detectably lower than in metameres Tr9 and Tr10 (see above). Regional transcriptional specialization in the tracheal system has already been demonstrated [41]. Further, the Tr2 regions of the DTs, which have the specialized role of generating the dorsal air sacs during pupal development, are distinctly different from other metameres in that their cells remain mitotically active and do not undergo polyploidization during larval life [42]. Studies of the tracheoblast cells associated with Tr4 and Tr5 have identified subpopulations of cells with different levels of mitotic activity differentiation, and polyploidization that are coordinated by a gradient of *cut* expression across each tracheoblast cell cluster [17, 43]. A precedent for *cut* regulation of regional specialization within the tracheal system therefore exits. However, other enhancers associated with the *cut* gene are known to regulate *cut* expression in the spiracles and tracheae [20, 44] and thus the aggregate expression pattern for *cut* in the DTs may differ considerably from that produced by the *cut(ue)* enhancer alone. Quantitation of Cut tracheal protein expression is needed to resolve this issue.

### Neural and non-neural roles for *lov* in behavior

Unlike larvae with *lov* tracheal knockdown, *lov^91Y^*-Gal4 hemizygous larvae show no indications of tracheal permeability but display backward movement, failure of systemic growth and poor survival to adulthood. These phenotypes are similar to those of *lov^47^*, a deletion mutation generated from the *lov^91Y^* chromosome in which 1.4 kb of DNA upstream of the *lov^91Y^* PGawB transposon insertion site are missing [6]. In *lov^47^* mutant embryos, Lov expression in many elements of the developing larval nervous system is lost, suggesting that the phenotypes generated are neural in origin [6]. It seems likely therefore that the *lov^91Y^*-Gal4 hemizygous larval phenotypes, like those of *lov^47^*, are also neural in origin.

The *lov^91Y^* PGawB insertion site is ~ 500 bp upstream of the start site for transcripts B and D of the *lov* gene [6]. Our previous studies with *lov^47^* indicated that the region around this site contains a regulatory element(s) controlling the expression of transcript D in neural tissues. The Gal4 expression pattern for the *lov^91Y^* PGawB insertion described here reveals the presence of additional regulatory sequences in this region that control *lov* expression in the larval tracheae. The tracheal phenotypes identified here demonstrate the effects of specific knockdown of this component of *lov* expression in larval life. However, the *lov^47^*and *lov^91Y^* -Gal4 phenotypes probably derive from direct disruption of neural regulatory elements within this same DNA region and thus affect neural roles for the gene in larvae.

The immediate stimulus for a behavioral response is typically neural. From the middle stages of embryogenesis until hatching, *lov* is expressed strongly and almost exclusively in the developing nervous system [6] and all the behavioral phenotypes we have previously identified for *lov* are probably neural in origin. In contrast, these current studies define an additional route by which *lov* may activate behavioral responses and in general, they underscore the fact that genes can influence behavior in multiple, often indirect, ways.

## Material and Methods

### Drosophila stocks

The following stocks were obtained from the Bloomington Drosophila Stock Center (BDSC); **BL27327** (*w-*; *cut*-Gal4.B - here called *cut(ue)-*Gal4); **BL8807** (w*; *btl*-Gal4, UASp-*Act*5C-GFP/*CyO*, PlacZ)**; BL4961**(Df (2R) *K10 b pr Bl c* (here called *lov* deficiency chromosome, *lov def*) /SM1); the *CyO*-GFP balancers from stocks **BL5702** (*Sco*/*CyO*, *hsp*-*70*-Gal4,UAS- GFP) and **BL6662** (*w-*; *Gla*/ *CyO twi*-Gal,UAS-2xEGFP) were used to prepare *lov* deficiency chromosome/*CyO*-GFP stocks. *lov* RNAi stocks used were as follows; **#10739**, from the Vienna Drosophila Resource Center; **#JF02205** (Valium 10 construct) and **#HMS01126** (Valium 20 construct) from the TRiP Project at Harvard Medical School. Canton-S was used as the wild type (+) stock for these studies. *lov^91Y^* was identified in a previous screen [5]. Stocks were used as received from suppliers.

### Burrowing and tunneling assay

Petri plates (10 cm) with a layer (3–4 mm) of 2% agarose were generated and 10 newly hatched larvae of a given genotype were placed in a central well containing a standard amount (0.8 gms) of yeast paste (7 gms yeast, 10 ml water). At least 50 larvae for each genotype were examined. Growth, burrowing and tunneling behavior, and survival were observed daily through to adulthood. Once adults had emerged, plates were washed extensively with water to remove debris and dried for 1–2 days. Images of the tunneling patterns on the plates were then taken with a Biorad GelDoc Universal Hood II system, using a black background. Under these conditions the tunnels appeared as white strips against the dark background. Tunneling was then quantitated from negatives of these images by calculating the total number of dark pixels in tunnels per plate using NIH image J. An initial round of studies used agarose plates without mold inhibitor. After noting that mold growth in the tunnels increased the pixel count on Image J, 0.25% methyl p-hydroxybenzoate (Nipagen) was included in all plates. Comparisons of tunneling activity were only performed between sets of plates that were either all + Nipagen or—Nipagen.

### Tracheal imaging and analysis

Embryos from the appropriate crosses were collected on grape plates for four hours and after hatching, larvae were raised on mounds of yeast paste on grape plates at 22oC. For crosses involving *btl*-Gal4, UAS-Actin-GFP/*CyO*, GFP larvae were selected using a Leica MX FluoIII fluorescence microscope. For imaging, larvae were immobilized either with ether vapor before mounting in 70% glycerol or by heating for a few seconds on a 70oC hot plate [26] after mounting. Microscopes used for imaging were as follows; bright field and fluorescence images in Fig 1 (A-D, F, G) and Fig 5 (G, H)—Zeiss Axioplan2; bright field images in Fig 5 (A-D)—Zeiss Axioimager 2; bright field images in Fig 1(E) and Fig 5 (E, F)—Zeiss Axioskop. For quantitation of tracheal defects (Fig 5), larvae were imaged from Day1 after hatching until the day before pupation or death, as determined form the studies in Figs 3 and 4. At least 10 larvae were imaged for each genotype and fluid-filling of the tracheae was quantitated from stored image sets.

### Larval Locomotion Assay

Embryos from appropriate crosses were collected on grape plates for four hours. The midpoint of this four hours was used as the Average Egg Lay time (AEL). Larvae were fed on mounds of yeast paste on grape plates at 22oC and assayed 90–92 hours after AEL. A *lov def* /*CyO twi*-2x GFP stock was used for crosses to generate hemizygous larvae and non-GFP larvae were selected at 72 hours AEL then reared separately to 90–92 hours AEL. For assay, each larva was transferred to a clean 2% agarose plate, given one minute to acclimatize and then forward strides, backward strides and head turns were scored over a one minute period under a dissecting microscope. Assays were performed at 18oC, >50 larvae of each genotype were analyzed. Student’s t-test was used to compare all other genotypes to the *cut(ue)*-Gal4/+ control.

### Semi-Quantitative RT-PCR

RNA was isolated from embryos or larvae using Trizol lysis as previously [45]. RNA was reverse transcribed with Superscript III reverse transcriptase (Invitrogen) and a random hexamer mix (New England Biolabs). PCR (30 cycles) at appropriate annealing temperatures was used to generate DNA fragments specific for each targeted gene. Agarose gel electrophoresis and GelRed Nucleic Acid Stain (Phenix) were used to detect DNA fragments. *Actin*57*B*, which is abundantly expressed in larvae, and ribosomal protein gene *rp49* were used as control genes for standardizing expression. Primers used were:

*actin* forward: 5’ TTCCAAGCCGTACACACCGTAACT 3’

*actin* reverse: 5’ TCATCACCGACGTACGAGTCCTTCT 3’

*rp49* forward: 5' TACAGGCCCAAGATCGTGAA 3'

*rp49* reverse: 5' CACGTTGTGCACCAGGAACT 3'

*lactate dehydrogenase* set 1 forward: 5’ CATCCTTGTCAATGCCATGTTC 3’

*lactate dehydrogenase* set 1 reverse: 5’ TGCTTATGGTGTCCAATCCC 3’

      set 2 forward: 5' CTGAAGAACCCCCAGATCAC 3'

      set 2 reverse: 5' GCAAAATGGTATCGGGACTG 3'

*branchless* forward: 5' TTGCCTGTATCTCTGCATGG 3'

*branchless* reverse: 5' TCGTGTAGGTGCTCAGCTTG 3'

*fatiga* forward: 5' GACAAGATCCGAGGCGATAA 3'

*fatiga* reverse: 5' CGCTCCCTGATGTGGTAGTT 3'

*dDPH1* forward: 5' TGCCAGAGACAACGAAGATG 3'

*dDPH1* reverse: 5' GCCTTGTCTTCCGAGTGTTC 3’

### Tracheal dissection

Larvae were dissected in phosphate buffered saline (PBS) with 1mM EDTA, following the protocol described by Ramachandran and Budnik [46]. The internal organs except the tracheal system were cleaned out as much as possible. Posterior spiracles were cut off without destroying the last segment of the tracheal system. After dissection, the entire tracheal system was still attached to the cuticle. Preparations were then fixed in 3% paraformaldehyde for 30 min. After washing with PBS, tracheae were ready for further treatment.

### Immunostaining

Fixed dissected larval tracheae were blocked in BBXN (PBS, 0.3% Triton-X 100, 0.1% BSA, 5% goat serum). Primary antibodies used were i) guinea pig anti-Lov, prepared in two guinea pigs (Cocalico Biologicals, Inc) against the Lov protein fragment used previously to generate rabbit and guinea pig Lov antisera [6, 47], and ii) mouse anti-Coracle and anti-Fas III both from the Developmental Studies Hybridoma Bank. Anti-Lov was used at a 1:50 dilution in BBXN, followed by biotin-labeled secondary antibody (Vector Labs, 1:500 dilution in BBXN), streptavidin-horse radish peroxidase (Thermo scientific), and metal-enhanced 3, 3’- Diaminobenzidine (DAB) (Thermo scientific) detection. Anti-Fas III and anti-Coracle were used at 1:10 or 1:5 dilution in BBXN) and detected with goat anti-mouse IgG labelled with Alexa Fluor 594 nm (BD BioSciences, 1:500 dilution in BBXN). Tracheae were detached from the larval cuticle before mounting. Images were taken with a Zeiss Axioskop or a Zeiss LSM 710 confocal microscope.

### Polyploidy and cell number measurements

Dissected tracheae were treated sequentially with 0.1% Triton in PBS for 10 min, 0.1M ammonium chloride in PBS for 10 min, 50 mg/μl RNAse A (Thermo scientific) in PBS for 1 hour and finally DAPI (4', 6-diamidino-2-phenylindole, 0.01 mg/ml in PBS, Invitrogen) for 5 min. Tracheae were then detached from the cuticle for mounting. To quantitate DAPI intensity, image series were taken via a Zeiss LSM 710 confocal microscope with a 40x oil immersion objective. Nikon NIS-Element C software was then used to build 3D images of nuclei and quantitate DAPI intensity for each selected nucleus. An average of six nuclei were analyzed in each tracheal segment, and tracheae from eight individual larvae were analyzed per genotype. To quantitate cell numbers in Tr9 and Tr10 of the tracheal system, a Zeiss Axioplan2 was used, and 10 individual tracheal pairs were analyzed for each genotype.

## Supporting Information

S1 Fig Comparison of *lov* knockdown with three different *lov* RNAi constructs. All the *lov* RNAi lines were driven by *elav*-Gal4. Embryos (12–16 hrs after egg laying) were collected for RNA extraction. Semi-Q RT-PCR was used for transcript quantification. *lov* and *actin* PCR products for each RNA sample were run in parallel in separate agarose gel lanes. RNA preparations from two sets of embryos gave identical results. See Material and Methods for RNAi line sources.
(TIF)

S2 Fig Quantitation of failed larval burrowing and death in various genotypes. Larvae were placed in tunneling assays one day after hatching as described in Material and Methods. (a) for both *cut(ue)-*Gal4 > *lov* RNAi and *btl*-Gal4 > *lov* RNAi larvae, failure to burrow (larvae outside the food) is seen but this is associated with greater larval death (as opposed to transition to pupation) for *btl*-Gal4 > *lov* RNAi larvae. (b) In the *lov* hemizygous condition, *cut(ue)-*Gal4/*lov def* > *lov* RNAi larvae outside the food die in greater numbers rather than pupating. Larvae were assayed in batches of 10. At least five batches per genotype were examined. Error bars = +/- SEM.
(TIF)

## References

[1] Rodriguez L, Sokolowski MB, Shore JS. Habitat selection by Drosophila melanogaster larvae. J Evol Biol. 1992;5: 61–70.

[2] Carton Y, Sokolowski M. Parasitization of embedded and nonembedded *Drosophila melanogaster* (Diptera: Drosophilidae) pupae by the parasitoid *Pachycrepoideus vindemniae* (Hymenoptera: Pteromalidae). J Insect Behav. 1994;7: 129–131.

[3] Wong JL, Sokolowski MB, Kent CF. Prepupation behavior in Drosophila: embedding. Behav Genet. 1985;15: 155–164. 3939400 10.1007/BF01065896

[4] Narasimha S, Kolly S, Sokolowski MB, Kawecki TJ, Vijendravarma RK. Prepupal building behavior in *Drosophila melanogaster* and its evolution under resource and time constraints. PLOS ONE. 2015;10: e0117280 10.1371/journal.pone.0117280 25671711 PMC4324899

[5] Armstrong JD, Texada MJ, Munjaal R, Baker DA, Beckingham KM. Gravitaxis in *Drosophila melanogaster*: a forward genetic screen. G2B. 2006;5: 222–239. 10.1111/j.1601-183X.2005.00154.x 16594976

[6] Bjorum SM, Simonette RA, Alanis R Jr, Wang JE, Lewis BM, Trejo MH, et al The Drosophila BTB domain protein Jim Lovell has roles in multiple larval and adult behaviors. PLOS ONE. 2013;8: e61270 10.1371/journal.pone.0061270 23620738 PMC3631165

[7] Albagli O, Dhordain P, Deweindt C, Lecocq G, Leprince D. The BTB/POZ domain: a new protein-protein interaction motif common to DNA- and actin-binding proteins. Cell Growth Diffn. 1995;6: 1193–1198. 8519696

[8] Brand A, Perrimon N. Targeted gene expression as a means of altering cell fates and generating dominant phenotypes. Development. 1993;118: 401–415. 8223268 10.1242/dev.118.2.401

[9] Bodmer R, Barbel S, Sheperd S, Jack JW, Jan LY, Jan YN. Transformation of sensory organs by mutations of the cut locus of *D*. *melanogaster*. Cell. 1987;51: 293–307. 3117374 10.1016/0092-8674(87)90156-5

[10] Jack J, Dorsett D, Delotto Y, Liu S. Expression of the cut locus in the Drosophila wing margin is required for cell type specification and is regulated by a distant enhancer. Development. 1991;113: 735–747. 1821846 10.1242/dev.113.3.735

[11] Jack J, DeLotto Y. Structure and regulation of a complex locus: the cut gene of Drosophila. Genetics. 1995;139: 1689–1700. 7789769 10.1093/genetics/139.4.1689 PMC1206494

[12] Guo Y, Livne-Bar I, Zhou L, Boulianne GL. Drosophila presenilin is required for neuronal differentiation and affects notch subcellular localization and signaling. J Neurosci. 1999;19: 8435–8442. 10493744 10.1523/JNEUROSCI.19-19-08435.1999 PMC6783002

[13] Wingrove JA, O'Farrell PH. Nitric oxide contributes to behavioral, cellular, and developmental responses to low oxygen in Drosophila. Cell. 1999;98: 105–114. 10412985 10.1016/S0092-8674(00)80610-8 PMC2754235

[14] Venken KJ, Schulze KL, Haelterman NA, Pan H, He Y, Evans-Holm M, et al MiMIC: a highly versatile transposon insertion resource for engineering *Drosophila melanogaster* genes. Nature Methods. 2011;8: 737–743. 21985007 10.1038/nmeth.1662 PMC3191940

[15] Pitsouli C, Perrimon N. Embryonic multipotent progenitors remodel the Drosophila airways during metamorphosis. Development. 2010;137: 3615–2624. 10.1242/dev.056408 20940225 PMC2964094

[16] Attrill H, Falls K, Goodman JL, Millburn GH, Antonazzo G, Rey AJ, et al FlyBase: establishing a Gene Group resource for Drosophila melanogaster. Nucl Acids Res. 2016;44(D1): D786–D792. 10.1093/nar/gkv1046 26467478 PMC4702782

[17] Pitsouli C, Perrimon N. The homeobox transcription factor cut coordinates patterning and growth during Drosophila airway remodeling. Science Signaling. 2013;6: ra12 10.1126/scisignal.2003424 23423438 PMC3982146

[18] Wieschaus E, Nusslein-Volhard C, Jurgens G. Mutations affecting the pattern of the larval cuticle in *Drosophila melanogaster*. Wil Roux Arch Dev Biol. 1984;193: 296–307. 10.1007/BF00848158 28305339

[19] Blochlinger K, Jan LY, Jan YN. Postembryonic patterns of expression of cut, a locus regulating sensory organ identity in Drosophila. Development. 1993;117:441–50. 8330519 10.1242/dev.117.2.441

[20] Bourbon HM, Gonzy-Treboul G, Peronnet F, Alin MF, Ardourel C, Benassayag C, et al A P-insertion screen identifying novel X-linked essential genes in Drosophila. Mech Dev. 2002;110:7 1–83. 10.1016/s0925-4773(01)00566-4 11744370

[21] Zelzer E, Shilo BZ. Cell fate choices in Drosophila tracheal morphogenesis. BioEssays. 2000; 22: 219–226. 10684581 10.1002/(SICI)1521-1878(200003)22:3<219::AID-BIES3>3.0.CO;2-A

[22] Takaesu NT, Johnson AN, Newfeld SJ. Posterior spiracle specific GAL4 lines: new reagents for developmental biology and respiratory physiology. Genesis. 2002;34: 16–18. 12324940 10.1002/gene.10109

[23] Liu L, Johnson WA, Welsh MJ. Drosophila DEG/ENaC pickpocket genes are expressed in the tracheal system, where they may be involved in liquid clearance. Proc Natl Acad Sci USA. 2003;100: 2128–2133. 12571352 10.1073/pnas.252785099 PMC149970

[24] Morton DB. Behavioral responses to hypoxia and hyperoxia in Drosophila larvae. Fly. 2011;5: 119–125. 21150317 10.4161/fly.5.2.14284 PMC3127060

[25] Harrison JF, Haddad GG. Effects of oxygen on growth and size: synthesis of molecular, organismal, and evolutionary studies with Drosophila melanogaster. Annu Rev Physiol. 2011;73: 95–113. 10.1146/annurev-physiol-012110-142155 20936942

[26] Wong DM, Shen Z, Owyang KE, Martinez-Agosto JA. Insulin- and warts-dependent regulation of tracheal plasticity modulates systemic larval growth during hypoxia in *Drosophila melanogaster*. PLOS ONE. 2014;9: e115297 10.1371/journal.pone.0115297 25541690 PMC4277339

[27] Li Y, Padmanabha D, Gentile LB, Dumur CI, Beckstead RB, Baker KD. HIF- and non- HIF-regulated hypoxic responses require the estrogen-related receptor in *Drosophila melanogaster*. PLOS Genetics. 2013;9: e1003230 10.1371/journal.pgen.1003230 23382692 PMC3561118

[28] Tennessen JM, Bertagnolli NM, Evans J, Sieber MH, Cox J, Thummel CS. Coordinated metabolic transitions during Drosophila embryogenesis and the onset of aerobic glycolysis. G3 (Bethesda, Md). 2014;4: 839–850. 10.1534/g3.114.010652 PMC4025483 24622332

[29] Parvy JP, Napal L, Rubin T, Poidevin M, Perrin L, Wicker-Thomas C, et al *Drosophila melanogaster* Acetyl-CoA-carboxylase sustains a fatty acid-dependent remote signal to waterproof the respiratory system. PLOS Genetics. 2012;8: e1002925 10.1371/journal.pgen.1002925 22956916 PMC3431307

[30] Tepass U, Tanentzapf G, Ward R, Fehon R. Epithelial cell polarity and cell junctions in Drosophila. Annu Rev Genet. 2001;35: 747–784. 11700298 10.1146/annurev.genet.35.102401.091415

[31] Vermehren-Schmaedick A, Ainsley JA, Johnson WA, Davies SA, Morton DB. Behavioral responses to hypoxia in Drosophila larvae are mediated by atypical soluble guanylyl cyclases. Genetics. 2010;186: 183–196. 10.1534/genetics.110.118166 20592263 PMC2940286

[32] Church RB, Robertson FW. Biochemical analysis of genetic differences in the growth of Drosophila. Genet Res. 1966;7: 383–407. 5940873 10.1017/s0016672300009836

[33] Glasheen BM, Robbins RM, Piette C, Beitel GJ, Page-McCaw A. A matrix metalloproteinase mediates airway remodeling in Drosophila. Dev Biol. 2010;344: 772–783. 10.1016/j.ydbio.2010.05.504 20513443 PMC2914218

[34] Langlais KK, Stewart JA, Morton DB. Preliminary characterization of two atypical soluble guanylyl cyclases in the central and peripheral nervous system of Drosophila melanogaster. J Exp Biology. 2004;207: 2323–2338. 10.1242/jeb.01025 15159437

[35] Affolter M, Shilo BZ. Genetic control of branching morphogenesis during Drosophila tracheal development. Current Op Cell Biol. 2000;12: 731–735. 10.1016/s0955-0674(00)00160-5 11063940

[36] Ghabrial A, Luschnig S, Metzstein MM, Krasnow MA. Branching morphogenesis of the Drosophila tracheal system. Annu Rev Cell Dev Biol. 2003;19: 623–647. 14570584 10.1146/annurev.cellbio.19.031403.160043

[37] Uv A, Cantera R, Samakovlis C. Drosophila tracheal morphogenesis: intricate cellular solutions to basic plumbing problems. Trends Cell Biol. 2003;13: 301–309. 12791296 10.1016/s0962-8924(03)00083-7

[38] Smith AV, Orr-Weaver TL. The regulation of the cell cycle during Drosophila embryogenesis: the transition to polyteny. Development. 1991;112: 997–1008. 1935703 10.1242/dev.112.4.997

[39] Britton JS, Lockwood WK, Li L, Cohen SM, Edgar BA. Drosophila's insulin/PI3-kinase pathway coordinates cellular metabolism with nutritional conditions. Developmental cell. 2002;2: 239–249. 11832249 10.1016/s1534-5807(02)00117-x

[40] Sun J, Deng WM. Notch-dependent downregulation of the homeodomain gene cut is required for the mitotic cycle/endocycle switch and cell differentiation in Drosophila follicle cells. Development. 2005;132: 4299–4308. 16141223 10.1242/dev.02015 PMC3891799

[41] Faisal MN, Hoffmann J, El-Kholy S, Kallsen K, Wagner C, Bruchhaus I, et al Transcriptional regionalization of the fruit fly's airway epithelium. PLOS ONE. 2014;9: e102534 10.1371/journal.pone.0102534 25020150 PMC4097054

[42] Guha A, Lin L, Kornberg TB. Organ renewal and cell divisions by differentiated cells in Drosophila. Proc Nal Acad Sci USA 2008;105: 10832–10836. 10.1073/pnas.0805111105 PMC2504775 18664581

[43] Pitsouli C, Perrimon N. Embryonic multipotent progenitors remodel the Drosophila airways during metamorphosis. Development. 2010;137: 3615–3624. 10.1242/dev.056408 20940225 PMC2964094

[44] Nepveu A. Role of the multifunctional CDP/Cut/Cux homeodomain transcription factor in regulating differentiation, cell growth and development. Gene. 2001;270: 1–15. 11403998 10.1016/s0378-1119(01)00485-1

[45] Bogart K, Andrews J. Extraction of total RNA from Drosophila. Center for Genomics and Bioinformatics. 2006;CGB Technical Report 2006–10:doi:10.2506/cgbtr-200610.

[46] Ramachandran P, Budnik V. Immunocytochemical staining of Drosophila larval body- wall muscles. Cold Spring Harbor Protocols. 2010;pdb.prot5470. 10.1101/pdb.prot5470 20679379

[47] Bjorum SM. A lov story: a role for jim lovell in Drosophila neural development and fertility. PhD thesis, Rice University. 2010: 60–61.

