# Supplementary figures and images for: Failure to Burrow and Tunnel Reveals Roles for jim lovell in the Growth and Endoreplication of the Drosophila Larval Tracheae
Source: PLoS One. 2016 Aug 5;11(8):e0160233. doi: 10.1371/journal.pone.0160233 (PMC4975476; doi:10.1371/journal.pone.0160233)

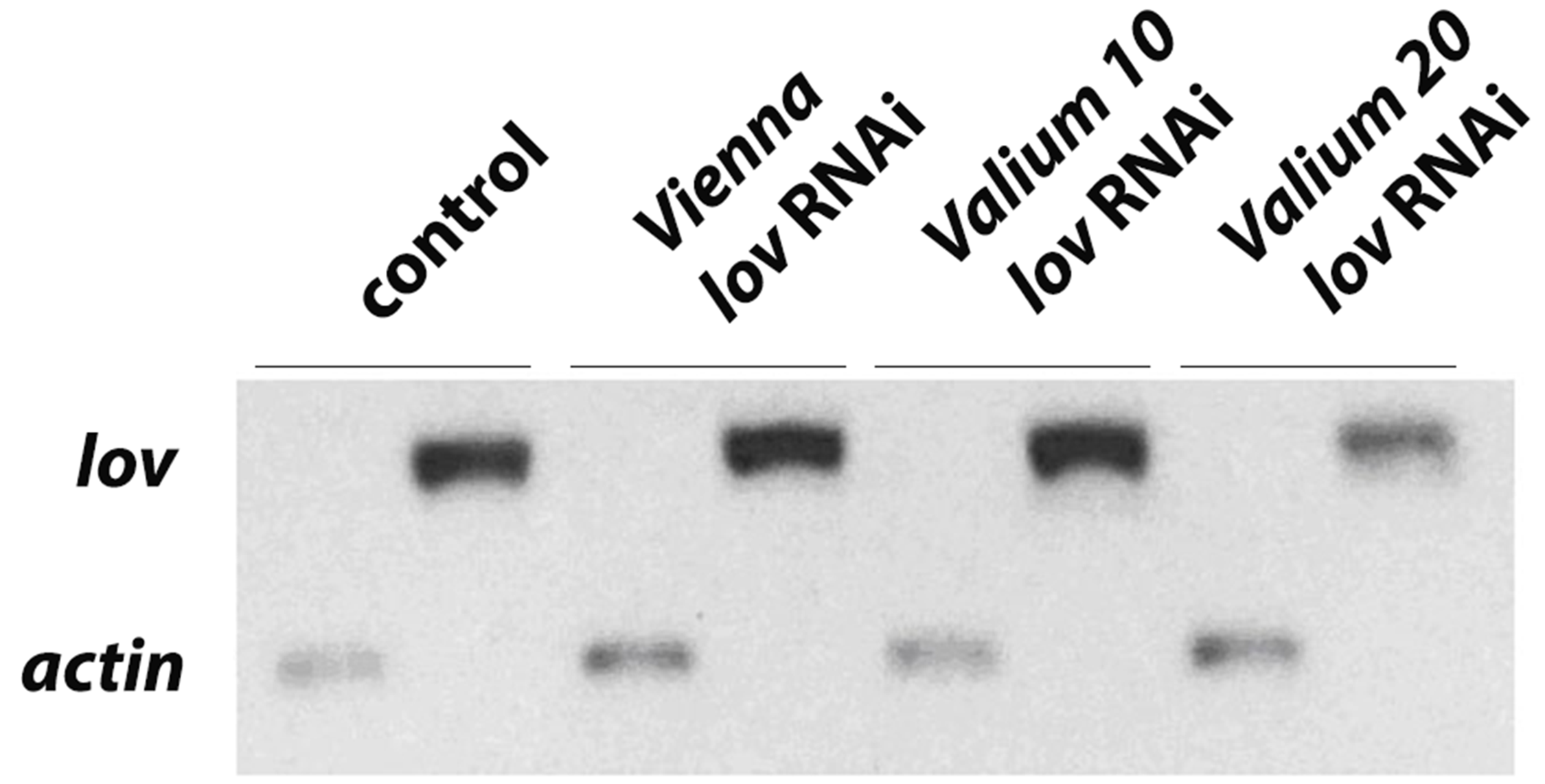

Supplement: S1 Fig — All the lov RNAi lines were driven by elav-Gal4. Embryos (12–16 hrs after egg laying) were collected for RNA extraction. Semi-Q RT-PCR was used for transcript quantification. lov and actin PCR products for each RNA sample were run in parallel in separate agarose gel lanes. RNA preparations from two sets of embryos gave identical results. See Material and Methods for RNAi line sources. (TIF) [file pone.0160233.s001.tif]

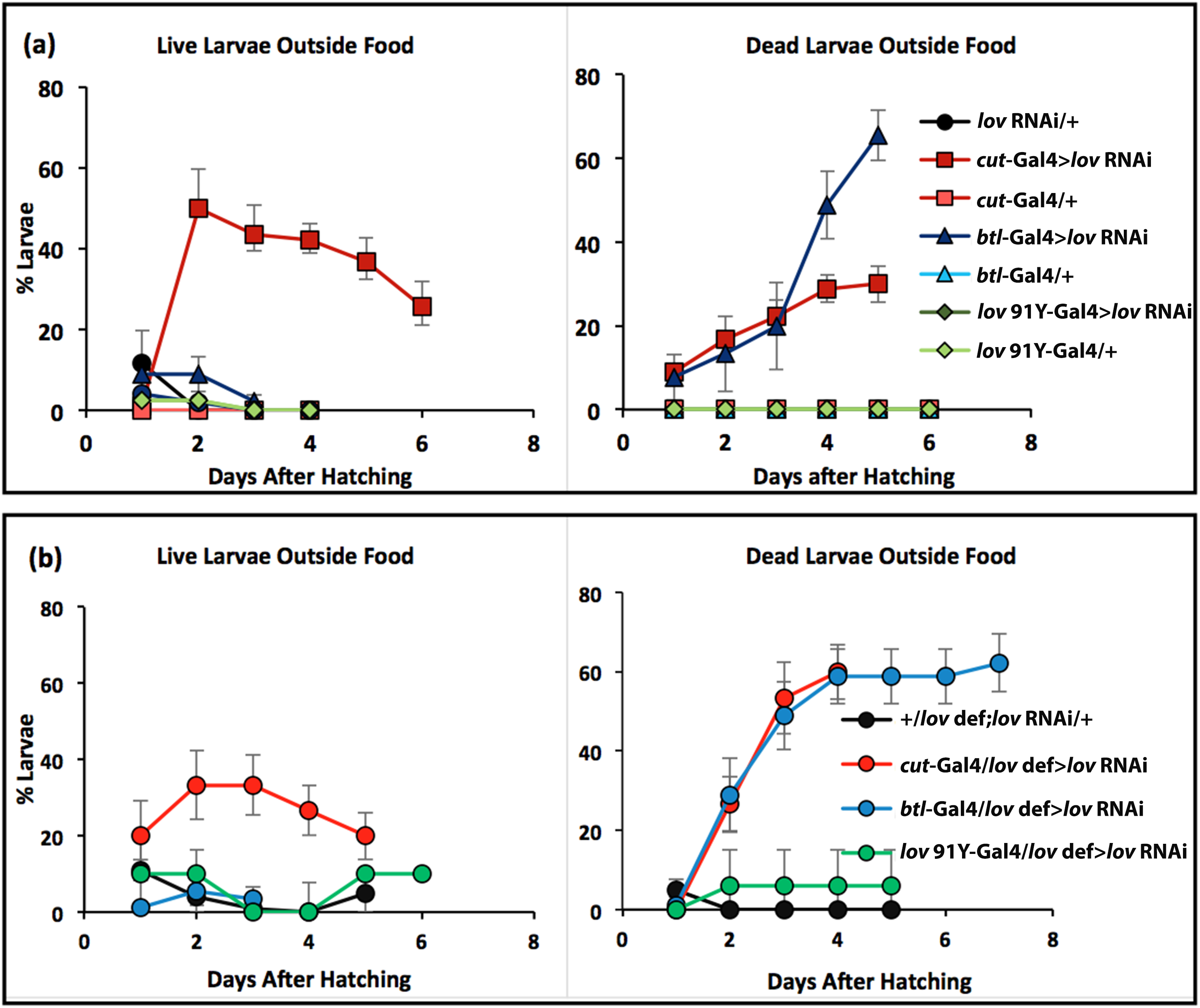

Supplement: S2 Fig — Larvae were placed in tunneling assays one day after hatching as described in Material and Methods. (a) for both cut(ue)-Gal4 > lov RNAi and btl-Gal4 > lov RNAi larvae, failure to burrow (larvae outside the food) is seen but this is associated with greater larval death (as opposed to transition to pupation) for btl-Gal4 > lov RNAi larvae. (b) In the lov hemizygous condition, cut(ue)-Gal4/lov def > lov RNAi larvae outside the food die in greater numbers rather than pupating. Larvae were assayed in batches of 10. At least five batches per genotype were examined. Error bars = +/- SEM. (TIF) [file pone.0160233.s002.tif]
